# The RNA of Maize Chlorotic Mottle Virus, an Obligatory Component of Maize Lethal Necrosis Disease, Is Translated via a Variant Panicum Mosaic Virus-Like Cap-Independent Translation Element

**DOI:** 10.1128/JVI.01005-20

**Published:** 2020-10-27

**Authors:** Elizabeth J. Carino, Kay Scheets, W. Allen Miller

**Affiliations:** a Department of Plant Pathology and Microbiology, Iowa State University, Ames, Iowa, USA; b Interdepartmental Genetics and Genomics Program, Iowa State University, Ames, Iowa, USA; c Department of Plant Biology, Ecology and Evolution, Oklahoma State University, Stillwater, Oklahoma, USA; University of California, Irvine

**Keywords:** 3′ CITE, *Tombusviridae*, long-distance base pairing, pseudoknot

## Abstract

Maize chlorotic mottle virus (MCMV) combines with a potyvirus in maize lethal necrosis disease (MLND), a serious emerging disease worldwide. To inform resistance strategies, we characterized the translation initiation mechanism of MCMV. We report that MCMV RNA contains a cap-independent translation element (CITE) in its 3′ untranslated region (UTR). The MCMV 3′ CITE (MTE) was mapped to nucleotides 4164 to 4333 in the genomic RNA. 2′-Hydroxyl acylation analyzed by primer extension (SHAPE) probing revealed that the MTE is a distinct variant of the panicum mosaic virus-like 3′ CITE (PTE). Like the PTE, electrophoretic mobility shift assays (EMSAs) indicated that eukaryotic translation initiation factor 4E (eIF4E) binds the MTE despite the absence of an m^7^GpppN cap structure, which is normally required for eIF4E to bind RNA. Using a luciferase reporter system, mutagenesis to disrupt and restore base pairing revealed that the MTE interacts with the 5′ UTRs of both genomic RNA and subgenomic RNA1 via long-distance kissing stem-loop interaction to facilitate translation. The MTE stimulates a relatively low level of translation and has a weak, if any, pseudoknot, which is present in the most active PTEs, mainly because the MTE lacks the pyrimidine-rich tract that base pairs to a G-rich bulge to form the pseudoknot. However, most mutations designed to form a pseudoknot decreased translation activity. Mutations in the viral genome that reduced or restored translation prevented and restored virus replication, respectively, in maize protoplasts and in plants. In summary, the MTE differs from the canonical PTE but falls into a structurally related class of 3′ CITEs.

**IMPORTANCE** In the past decade, maize lethal necrosis disease has caused massive crop losses in East Africa. It has also emerged in China and parts of South America. Maize chlorotic mottle virus (MCMV) infection is required for this disease. While some tolerant maize lines have been identified, there are no known resistance genes that confer immunity to MCMV. In order to improve resistance strategies against MCMV, we focused on how the MCMV genome is translated, the first step of gene expression by all positive-strand RNA viruses. We identified a structure (cap-independent translation element) in the 3′ untranslated region of the viral RNA genome that allows the virus to usurp a host translation initiation factor, eIF4E, in a way that differs from host mRNA interactions with the translational machinery. This difference indicates eIF4E may be a soft target for engineering of—or breeding for—resistance to MCMV.

## Abstract (precis)

In the past decade, maize lethal necrosis disease has caused massive crop losses in East Africa. It has also emerged in China and parts of South America. Maize chlorotic mottle virus (MCMV) infection is required for this disease. While some tolerant maize lines have been identified, there are no known resistance genes that confer immunity to MCMV. In order to improve resistance strategies against MCMV, we focused on how the MCMV genome is translated, the first step of gene expression by all positive-strand RNA viruses. We identified a structure (cap-independent translation element) in the 3′ untranslated region of the viral RNA genome that allows the virus to usurp a host translation initiation factor, eIF4E, in a way that differs from host mRNA interactions with the translational machinery. This difference indicates eIF4E may be a soft target for engineering of—or breeding for—resistance to MCMV.

## INTRODUCTION

Maize lethal necrosis disease (MLND, also referred to as corn lethal necrosis), first identified in the Americas in the 1970s (1), spread worldwide in the 2010s causing devastating crop losses and food insecurity across East Africa, where maize is the most important subsistence and cash crop (2–9). It has also emerged in China (10), Taiwan (11), Spain (12), and Ecuador where, in 2015 to 2016, the severe losses caused a state of emergency to be declared (13, 14).

MLND is caused by a mixed infection of maize chlorotic mottle virus (MCMV) and any potyvirid that infects maize, usually sugarcane mosaic virus (SCMV) (1, 15, 16). However, MCMV infection with viruses outside the *Potyviridae* family (9), or in the presence of abiotic stresses such as drought, can also be severe (5), while common maize potyviruses like SCMV are generally milder on their own (15, 17, 18). Efforts to identify genetic resistance against MCMV and potyviruses have revealed resistance to SCMV (19–21), but genes conferring resistance to MCMV have been elusive, despite much progress (22, 23) with reduced symptoms and virus levels. To our knowledge, no genes that confer complete resistance to MCMV have been identified. Despite its economic importance (5, 24–26), little is known about the molecular mechanisms of MCMV replication, gene expression, or its interactions with the host, which could provide valuable knowledge toward identifying targets for resistance breeding or engineering strategies.

**FIG 1:**
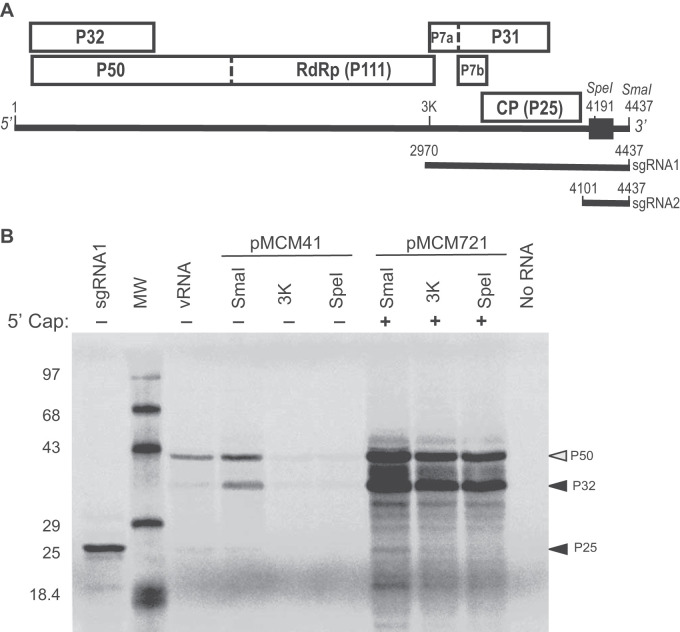
*In vitro* translation of MCMV genomic RNA. (A) Genome organization of MCMV. Boxes indicate open reading frames (ORFs) with the encoded proteins (named by the molecular weight in kDa) indicated. Dashed boundaries indicate leaky stop codons. RdRp, RNA-dependent RNA polymerase domain of P111; CP, coat protein. Positions of key restriction enzymes are indicated (SmaI: 4437; SpeI: 4191). Black box indicates the location of the 3′ CITE (nt 4164 to 4333). (B) Translation of capped (+) and uncapped (−) transcripts and virion RNA. pMCM41 was linearized at the indicated locations in the genome prior to transcription. Uncapped transcript from SmaI-linearized pMCM41 is infectious (29). Capped transcripts were from pMCM721, which is identical to pMCM41 but with one nonviral G at the 5′ end, which was required for capping. sgRNA1 is the uncapped transcript from psgRNA1 linearized with SmaI. Gel shows ^35^S-met-labeled products translated in WGE. The prominent bands identified on the gel correspond to P32, P50, and P25 products.

MCMV is the sole member of genus *Machlomovirus* in the family *Tombusviridae* (27). The 4437-nucleotide (nt) positive-sense RNA genome contains no 5′ cap, no poly(A) tail, and encodes seven open reading frames (ORFs) (28–30). The 5′ end of the genome contains two overlapping ORFs that code for a 32-kDa protein (P32) and a 50-kDa (P50) replicase-associated protein (RAP). The P50 ORF has a leaky stop codon which allows for readthrough translation of a 61-kDa C-terminal extension on P50 to form the 111-kDa RNA-dependent RNA polymerase (RdRp) (31) (Fig. 1A). In infected cells, MCMV generates two 3′-coterminal subgenomic RNAs that are 5′-truncated versions of the genomic RNA. Subgenomic RNA1 (sgRNA1), spanning nt 2971 to 4437, serves as mRNA from which the coat protein (CP), and the movement proteins P7a, P7b, and P31 are translated. The 337-nt sgRNA2, representing the 3′ untranslated region (UTR), is a noncoding RNA (28). Although the MCMV genome has been characterized to some extent (17, 28, 31), little is known about its translation mechanisms, a key process in the replication cycle.

Many positive-strand RNA viruses use noncanonical translation mechanisms, including cap-independent translation. This frees the virus from having to encode capping enzymes, and also allows the virus to avoid the host’s translational control system, which often acts through cap-binding proteins (32–34). Because it differs from host mechanisms, this virus-specific translation mechanism may provide unique targets for antiviral strategies. A translation strategy used by all studied tombusvirids is to harbor a cap-independent translation element (CITE) in the 3′ UTR of the virus’ genomic RNA, which is uncapped (35–37). The 3′ CITE replaces the role of the m^7^GpppN cap structure present at the 5′ end of all eukaryotic mRNAs. About seven different structural classes of 3′ CITE are known (35, 38, 39). Most 3′ CITEs attract the key ribosome-recruiting eukaryotic translation initiation factor heterodimer, eIF4F, by binding to one or both of its subunits, eIF4G and/or eIF4E (35, 39–43).

Because all other tested tombusvirids harbor a 3′ CITE, we predicted that MCMV RNA harbors a 3′ CITE to facilitate its translation. *In silico* analysis of the MCMV 3′ UTR using MFOLD and ViennaRNAfold to predict RNA secondary structures did not reveal a structure resembling a known 3′ CITE. Here, we provide experimental data that demonstrate the presence and function of a 3′ CITE that we call the MCMV 3′ CITE (MTE). The MTE is structurally similar to the panicum mosaic virus-like translation element (PTE) class of CITE, but with some key differences. We identify a key translation initiation factor with which the MTE interacts (eIF4E) and show how the MTE base pairs to the 5′ UTR to facilitate cap-independent translation, and that the functional MTE and the long-distance interaction are required for infection of maize. The results contribute to our understanding of structure-function relationships of cap-independent translation elements, and provide valuable information on the first step of gene expression for an important pathogen.

## RESULTS

### Mapping the 3′ cap-independent translation element in MCMV.

To roughly map the 3′ CITE of MCMV, we translated 3′-truncated transcripts from a full-length cDNA clone of the MCMV genome (pMCM41 [29]). Linearized pMCM41 DNA template was transcribed in the absence of cap analog, while pMCM721, which has a nonviral G preceding the 5′ terminal A of the MCMV genome, was used for capped transcripts because the 5′ A of pMCM41 (identical to MCMV RNA) could not be capped using an m^7^GpppA cap analog and T7 RNA polymerase (K.S., unpublished observation). Plasmid psgRNA1 was the template for transcription of full-length sgRNA1, the mRNA for the 25-kDa CP and the movement proteins (28). Transcribed RNAs and RNA isolated from virions (vRNA) were translated in wheat germ extract (WGE) in the presence of ^35^S-methionine. The full-length, infectious transcript from SmaI-linearized pMCM41 and vRNA yielded two protein products, P32 and P50, from the 5′-proximal overlapping ORFs (Fig. 1B). Interestingly, vRNA yielded much less P32 protein, relative to P50, than did the transcribed mRNA. Also, a faint band comigrating with CP is visible from both vRNA and the full-length transcript. The expected 111-kDa protein generated by readthrough of the P50 stop codon was not detected, most likely because ribosomal readthrough occurs at a very low rate under these conditions. Readthrough products have been difficult to detect among the *in vitro* translation products of other tombusvirid genomes as well (44–46). Unlike the full-length genomic RNA from SmaI-cut pMCM41, which yielded substantial protein products, the uncapped 3′-truncated transcripts produced almost no detectable protein product, suggesting that at least part of the 3′ CITE is downstream of the SpeI site at nt 4191 (Fig. 1B). It is noteworthy that translation in the presence of a 5′ cap on full-length and truncated pMCM721-derived RNAs gave much more translation product than uncapped full-length pMCM41 transcript or vRNA, indicating that the viral genome may be a relatively inefficient mRNA.

**FIG 2:**
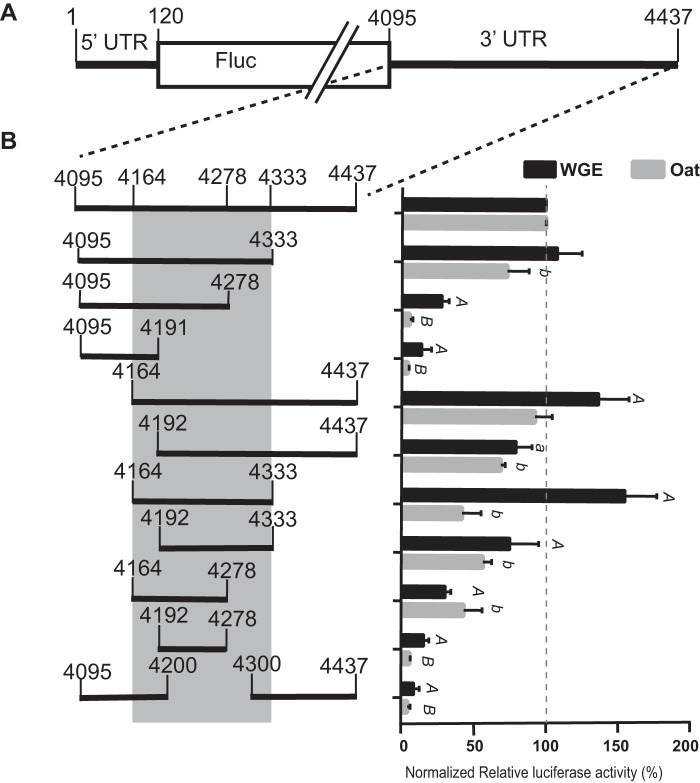
Mapping the sequence in MCMV 3′ UTR required for cap-independent translation. (A) Map of MlucM transcript containing a firefly luciferase reporter gene (Fluc) flanked by the complete MCMV UTRs, with base numbering as in the full-length genome. (B) Black bars below (left) indicate the portions of the 3′ UTR present in each MlucM deletion construct. Sequence covering nt 4164 to 4333 (gray shading) shows the minimal amount of sequence required for efficient cap-independent translation. Relative luciferase activity obtained from the indicated uncapped transcripts in WGE and oat protoplasts is shown (right) as percentages of relative light units where constructs were normalized to MlucM wild type 3′ UTR (top bar). Data are shown as averages, relative to full-length 3′ UTR (± standard deviation [SD]), from 4 independent experiments (for each construct: WGE, *n* = 10; oat protoplasts, *n* = 14). Protoplasts were coelectroporated with the indicated uncapped transcript plus capped *Renilla* luciferase mRNA as an electroporation control. Fluc/Rluc sample ratios were normalized to MlucM_wt_. One-way ANOVA was used to analyze the significance of each set of samples. For Dunnett’s multiple-comparison test significance, uppercase type (A or B) was assigned to a construct if the difference between MlucMwt and deletion mutant was *P* < 0.001, whereas lowercase type (a or b) was given to *P* < 0.05. “A or a” was assigned to data comparisons against MlucMwt in WGE, while “B or b” was assigned to comparisons in oat protoplasts.

**FIG 3:**
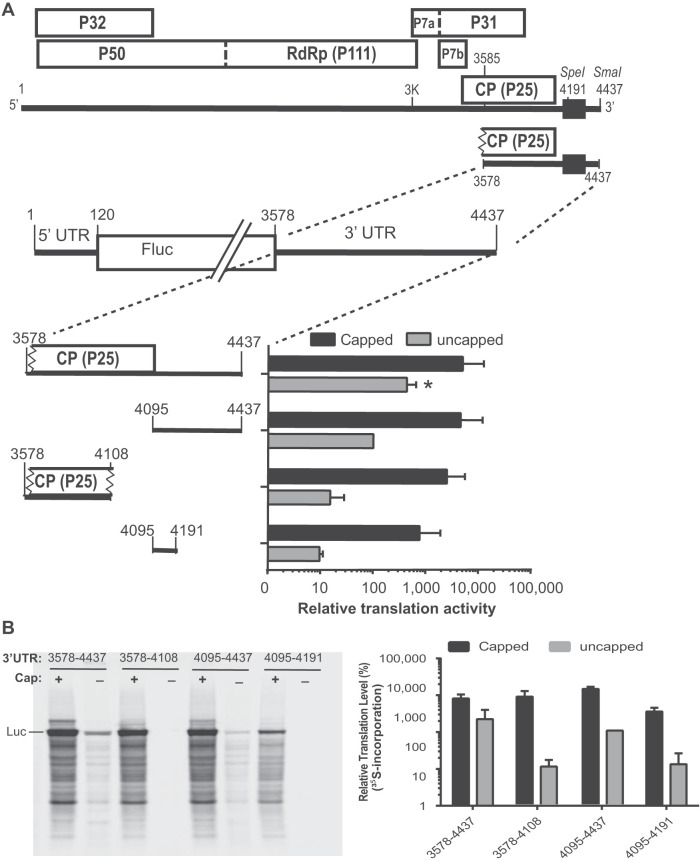
Luciferase translation assay, including the 3′ end of the coat protein coding region. (A) Genome organization of MCMV. Boxes indicate open reading frames (ORFs) with encoded proteins (named by the molecular weight in kDa) indicated. Black box shows location of the 3′ CITE (4164 to 4333). The zigzag lines indicate the truncated sequences of the CP ORF included in the luciferase construct. Luciferase translation assay results in WGE are shown to the right of the corresponding luciferase construct with the indicated 3′ UTR sequence. All constructs here contain the full MCMV 5′ UTR. Luciferase activities (relative light units) were normalized to the MlucM construct containing the full 3′ UTR (4095 to 4437). Data samples were subjected to one-way ANOVA and Dunnett’s multiple-comparison test was used to analyze the significance of each set of samples against MlucM, where one asterisk indicates a *P* value of 0.015. (B) *In vitro* translation assays using ^35^S-methionine. Left: a representative ^35^S-met-labeled gel sample out of 3 independent assays. Right: quantification of the overall radioactivity detected in each assay.

To rapidly map the 3′ CITE location at high resolution, a luciferase reporter (MlucM) was constructed such that the coding region of the virus was replaced by the firefly luciferase (Fluc) coding sequence (Fig. 2A). Deletion analyses showed little decrease in translation *in vitro* or *in vivo* when either the 3′-terminal 104 nt (nt 4334 to 4437) or the first 169 nt (nt 4095 to 4263) of the 3′ UTR were deleted (Fig. 2B). Additional constructs that included the adjacent sequence upstream of the 3′ UTR (517 nt of the CP ORF), up to nt 3578 in the MCMV genome, translated more efficiently than those that contained only the 3′ UTR (Fig. 3). However, the sequence upstream of the 3′ UTR (3578 to 4108) alone was not enough to support translation, and the greatest contributor to translation mapped to the 3′ UTR. Numerous deletions in the MCMV 3′ UTR revealed that the region between nucleotides 4164 and 4333 produced luciferase activity >100% of that from the full-length 3′ UTR *in vitro* and about 50% *in vivo*. The lower level of translation *in vivo* may be due to reduced RNA stability owing to the absence of the 3′ end, which is thought to confer stability in related viruses because of its highly base-paired terminal bases (47–49). Deletions within nt 4164 to 4333, especially of nt 4200 to 4300, reduced luciferase translation *in vitro*; thus, in subsequent studies we focused on nt 4164 to 4333 to characterize the MCMV 3′ CITE (MTE).

### Determining the secondary structure of MCMV 3′-CITE.

**FIG 4:**
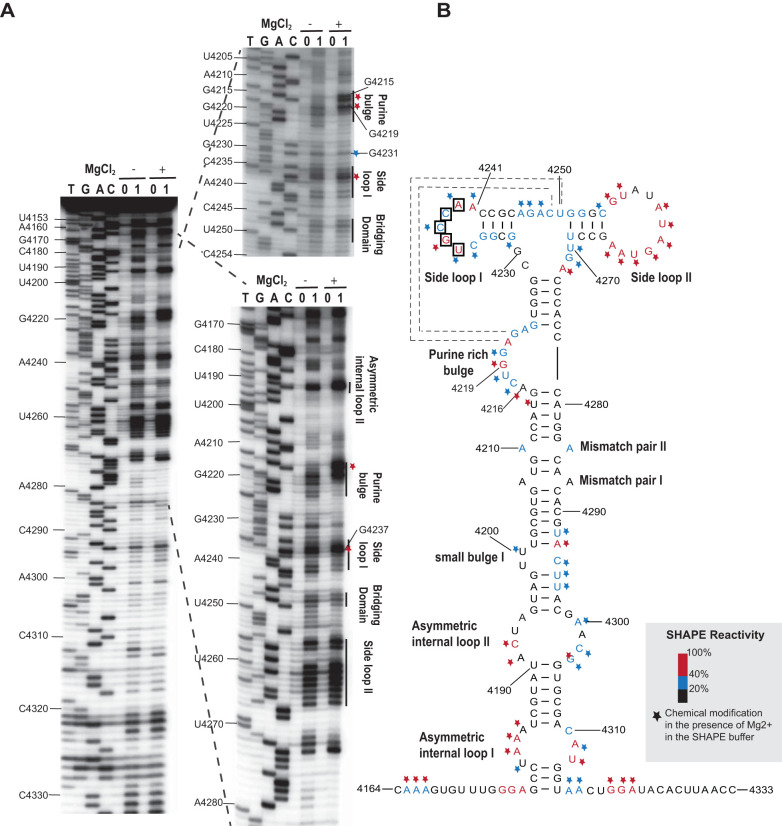
SHAPE analysis of the MTE structure from the MCM41 infectious clone. (A) SHAPE (selective 2′-hydroxyl acylation analyzed by primer extension) probing gel. RNA was modified with either 60 mM benzoyl cyanide (1) in dimethyl sulfoxide (DMSO) or DMSO only (0) in the presence (+) or absence (−) of magnesium in SHAPE buffer (see the Materials and Methods for details.) Gels show the products of reverse transcriptase extension of ^32^P-labeled primer on SHAPE-modified (1) and unmodified (0) templates. The sequencing ladders (four lanes at left, TGAC) were generated by dideoxy sequencing of unmodified RNA with the same 5′-labeled primer used in the modification lanes. Mobilities of selected nucleotides, numbered as in the viral genome, are shown at left. (B) Superposition of the degree of modification of each nucleotide on the best-fitting predicted secondary structure of MTE. Relative benzoyl cyanide modification is indicated by the color-coding scheme of the SHAPE radioactivity scale, as indicated at the lower right. Nucleotides inside boxes are suspected to base pair with the 5′ UTR. The modification levels of nucleotides in the presence of magnesium are indicated by stars following the reactivity color-coding scheme. Potential pseudoknot interactions between the purine-rich bulge and the bridging domain are indicated by dashed lines. Note: products of primer extension inhibition obtained from the SHAPE reaction are 1 nt shorter than those of dideoxy sequencing.

We determined the secondary structure of the MTE (nt 4164 to 4333) experimentally by subjecting it to selective 2′-hydroxyl acylation analyzed by primer extension (SHAPE) probing (Fig. 4A). This revealed that the sequence spanning nt 4166 to 4329 forms a long helical region with various asymmetric internal loops and bulges topped by two branching stem loops (Fig. 4B), which differed from the structures predicted using MFOLD (50) and the ViennaRNA package (51). This 164-nt secondary structure appears to be longer than those of any known PTEs. The main stem contains a purine-rich bulge between nucleotides 4216 to 4223. In the presence of magnesium ion, bases G_4215_, A_4216_, and G_4219_ were highly modified by the SHAPE reagent benzoyl cyanide, while bases AGA_4221–4223_ became less modified (Fig. 4A). The MTE also contains a single-stranded “bridging domain” (nt 4246 to 4250) connecting the two branching stem-loops, which was moderately modified in the presence and absence of magnesium. Side loop-I (SL-I_4235–4241_) houses a pentamer, UGCCA_4236–4240_ (bases in boxes, Fig. 4B), in its loop that is complementary to sequence UGGCA in the 5′-UTR. These pentamers may create a long-distance base-pairing interaction between the 3′ and 5′ UTRs (discussed later). The overall structure obtained from the SHAPE probing assays indicated that the MTE has a similar structure to those of panicum mosaic virus-like 3′ CITES (PTEs) (38, 52), but differing by the presence of three highly modified bases rather than a single hypermodified G in the purine-rich bulge in the presence of Mg^2+^ (53).

### Comparison of the MTE to PTEs: role of the pseudoknot.

**FIG 5:**
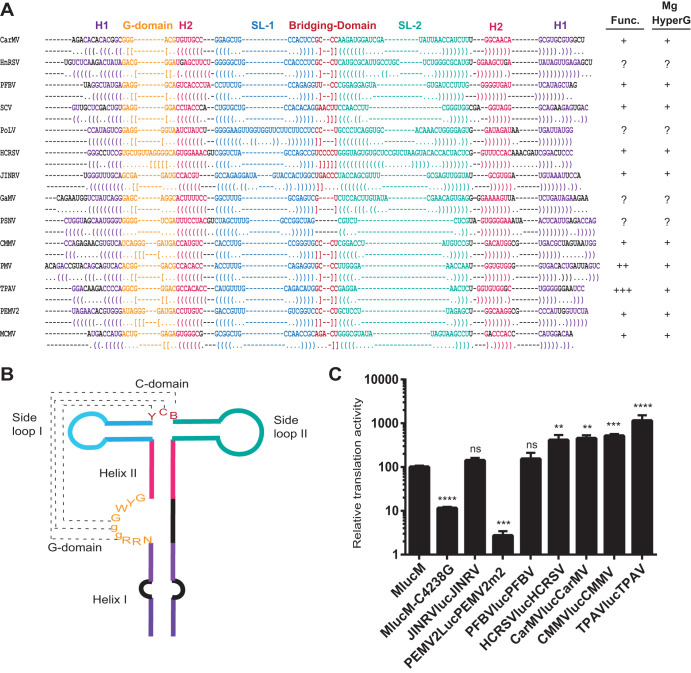
Alignment of known and predicted PTEs. (A) Secondary RNA structure alignment of PTEs. Structural input for alignment was obtained from previously published data and our predictions for more recently published viral sequences (Fig. S1 in the supplemental material). LocARNA (55) was used to identify conserved regions of the PTE structure, after which structures were aligned to fit the consensus. Bases are color coded based on the specific structure in the PTE, as sketched in (B) and explained as follows: purple: conserved helix I region (H1); orange, conserved purine rich bulge; magenta, conserved helix II region (H2); blue, conserved stem-loop I (SL1); red, bridging domain; and green, conserved stem-loop II (SL2). In the alignment, parentheses or square brackets of the same color in opposite orientation are below complementary bases. Square brackets show potential pseudoknot base pairing. (B) Sketch of conserved consensus shape of PTEs. Color coding corresponds to the alignment results in (A), as explained above. Conserved bases are shown using IUPAC nomenclature (Y = C or U; R = A or G; W = A or U; and B = all except A). The lower case g indicates G in ≥78% of PTEs. (C) Relative translational activity of different PTEs in WGE with wild-type MCMV indicated as 100%. Previously constructed luciferase reporter constructs contain a firefly luciferase reporter gene flanked by 5′ and 3′ UTRs of the indicated plant viral genomes containing PTEs in the 3′ UTR (56). PEMV2-m2 contains a CC to AA mutation in the bridging domain. Uncapped RNA transcripts were incubated for 30 min in WGE at room temperature. Data shown are percentage averages (±SD) from 3 independent experiments (*n* = 9), with two, three, and four asterisks indicating *P* < 0.01, *P* < 0.001, or *P* < 0.0001, respectively, for significance of difference from the wild-type MCMV (MlucM) construct. ns, not significantly different from MCMV.

Because the MTE SHAPE probing experiments suggested that the MTE resembled a PTE, we compared the secondary structure of the MTE with known and predicted PTEs using the alignment program LocARNA (54, 55) (Fig. 5A). This alignment revealed a consensus structure with more variability than reported previously (53), because more predicted PTE sequences are aligned than previously. The MTE and PTEs contain a purine-rich bulge with at least one highly conserved G. However, the previously termed “C-rich” domain of PTE that bridges between stem-loops 1 and 2 is not always C-rich, thus we now call it the bridging domain. One putative PTE, from pea stem necrosis virus (PSNV), contains no bridging domain and only a two-base-pair stem in stem-loop 2 (Fig. 5A). However, it has not been demonstrated to be functional. Potential pseudoknot base pairing between the purine-rich bulge and the bridging domain (square brackets, Fig. 5A) can be drawn for all PTEs except PSNV. However, for the MTE and some other PTE-like structures, the pseudoknot, if it exists at all, would consist of only two Watson-Crick base pairs: AG_4221–4222_:CU_4249–4250_ in the MTE. The SHAPE probing (Fig. 4A) indicates that modification of AG_4221–4222_ decreased in the presence of Mg^2+^ (which favors pseudoknot formation), and they are thus probably base-paired. Yet, the already-modest SHAPE sensitivity of bases CU_4249–4250_ in the bridging domain does not decrease in the presence of Mg^2+^, as would be expected if the proposed pseudoknot forms. The bridging domains of other PTEs in which this pseudoknot is likely, however, also show little change in modification in the presence of Mg^2+^ (53). Thus, as with other PTEs, although phylogenetic and structural data suggest this pseudoknot occurs, we cannot conclude this without doubt.

Because the MTE at least partially resembles the PTE consensus, we compared the MTE translation stimulation activity with that of other PTEs. Translation activity of luciferase reporter constructs containing PTEs in the 3′ UTR and the corresponding viral 5′ UTR (56) were compared to a construct containing MCMV 5′ and 3′ UTRs (MlucM). MlucM stimulated translation at a low level relative to most PTEs (Fig. 5C). However, it stimulated translation 9-fold more than the MCMV mutant C4238G, which prevents base pairing of the MTE to the 5′ UTR (see below), and 20-fold more than the negative control PEMV2lucPEMV2m2 (containing a CC-to-AA mutation in the bridging domain), which was shown previously to virtually eliminate PTE activity (53, 56). As reported previously (56), the PTE of thin paspalum asymptomatic virus (TPAV) stimulated translation to the highest level. The PTEs of Japanese iris necrotic ring virus (JINRV) and Pelargonium flower break virus (PFBV) were not significantly more stimulatory of translation than that of MCMV. Thus, even though the MTE resembles the PTE structure, it appears that MCMV (and JINRV and PFBV) have relatively weak PTE-like 3′ CITEs compared to other characterized PTEs. However, additional translation-enhancing sequences may reside in coding regions of these viruses. It is noteworthy that here and previously (56), the most stimulatory PTEs (TPAV and PMV) have strong GGG:CCC pseudoknot base pairing between the purine-rich bulge and the bridging domain, whereas “weak” PTEs, such as the MTE and JINRV, have little, if any, pseudoknot base pairing (Fig. 4B and Fig. S1 in the supplemental material, respectively). The role of potential pseudoknot base pairing is explored further below.

**FIG 6:**
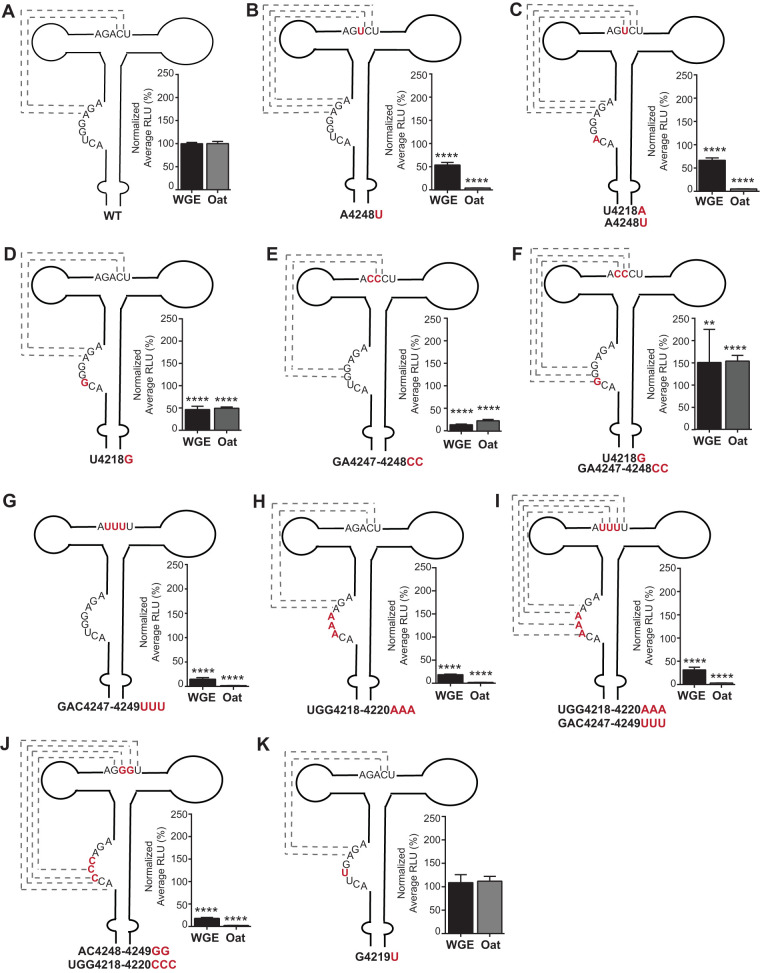
Effect on translation of MTE mutations in the potential pseudoknot interaction region. (A to K) Predicted structures of pseudoknot interaction mutants. Mutated bases in the MTE of each MlucM construct are in red. Constructs are named for the base changes at the numbered positions. Potential pseudoknot interactions are marked by dashed lines, with G:U pairs in a lighter shade. Relative luciferase translation activities in WGE (black bars) or oat protoplasts (gray bars) are shown in percentages of relative light units normalized to MlucM wild type. Data are averages (±SD) from 4 independent experiments (*n* = 15). Fluc/Rluc sample ratios were normalized to MlucM wt. One-way ANOVA was used to analyze the significance of each set of samples. One-way ANOVA-Dunnett’s multiple-comparison test was performed to compare statistical difference among double mutants and single mutants. Four asterisks indicate statistical difference with *P* < 0.0001; two asterisks indicate statistical difference with *P* < 0.005.

We constructed a series of mutations in the purine-rich bulge and bridging domain to test whether changes in these areas predicted to strengthen or weaken the pseudoknot had effects on translation efficiency (Fig. 6). These included mutations designed to determine if a stronger pseudoknot could increase translation activity. Mutant A4248U, which should lengthen the potential wild-type pseudoknot base pairing from two (AG_4221–4222_:CU_4249–4250_) to three (AGA_4221–4223_:UCU_4248–4250_) base pairs, translated only 55% as efficiently as wild type in WGE (Fig. 6B). This mutant could also potentially form an ACU_4216–4218_:AGU_4246–4248_ pseudoknot helix. To disrupt that possibility, a U4218A mutation was added. This double mutant translated 70% as efficiently as wild type in WGE (Fig. 6C). However, neither of these mutants translated appreciably in the more competitive conditions in protoplasts. In other constructs, mutations in both the purine-rich bulge and the bridging domain were introduced to generate pseudoknot base pairing predicted to be more stable than wild type. In constructs in which the purine-rich bulge remained purine-rich and the bridging domain became pyrimidine-rich, changing the purine-rich domain or the bridging domain alone reduced translation (Fig. 6D, E, G, and H), while the double mutants capable of forming the pseudoknot (GGG:CCC or AAAA:UUUU) translated more efficiently than the single-domain mutants. The GGG:CCC pseudoknot actually yielded 50% more luciferase than wild type in WGE and protoplasts (Fig. 6F), whereas the AAAA:UUUU predicted pseudoknot translated 35% as efficiently as wild type in WGE (Fig. 6I), which was slightly greater than the UUU mutation alone (which may form a weak pseudoknot containing two G:U pairs) or the AAA mutation in the purine-rich bulge. Each of these mutants translated about 15 to 20% as efficiently as wild type in WGE. However, none from this set of mutants translated detectably in protoplasts (Fig. 6G to I). Swapping the purines and pyrimidines to create a potential ACCC:GGGU pseudoknot helix gave low and no cap-independent translation in WGE and protoplasts, respectively (Fig. 6J). One mutation, G4219U in the purine-rich bulge, was not predicted to affect pseudoknot interactions and did not affect translation activity of the MTE (Fig. 6K). This is interesting because G4219 is highly modified in the presence of Mg^2+^ (Fig. 4). Overall, with one rather modest exception (Fig. 6F), mutations designed to increase pseudoknot base pairing altered the structure in such a way as to decrease translation efficiency.

### MTE binds eIF4E.

**FIG 7:**
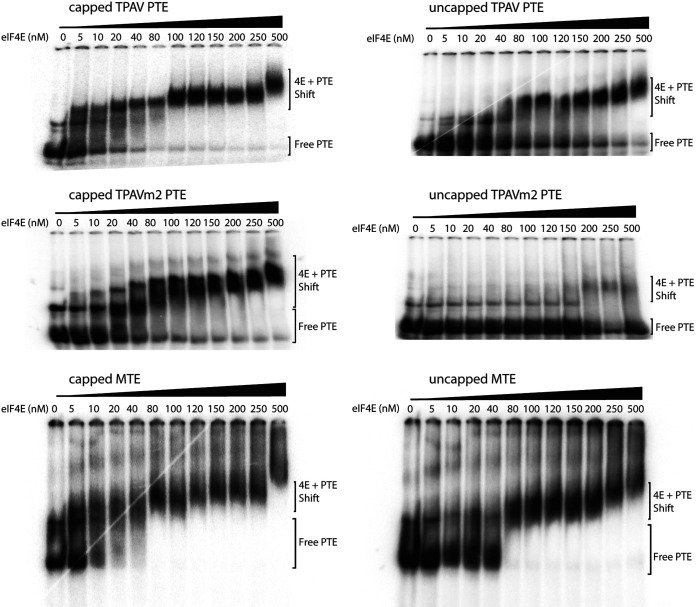
Electrophoretic mobility shift assays (EMSAs) of PTE and MTE RNA with eIF4E. The indicated capped or uncapped ^32^P-labeled transcripts (10 fmol) were incubated with the indicated concentrations of wheat eIF4E prior to electrophoresis on a nondenaturing gel. See the Materials and Methods for details.

Previously, PTEs have been shown to bind and require eukaryotic translation initiation factor 4E (eIF4E, the cap-binding protein), despite the absence of methylation (cap structure) on the PTE RNA (53, 57). Because the MTE resembles PTEs, we used electrophoretic mobility shift assays (EMSAs) to determine whether the MTE also binds eIF4E in the absence of a 5′ cap. To confirm integrity (cap-binding ability) of eIF4E, capped forms of all tested constructs were incubated in the presence of eIF4E and shown to confer strong mobility shifts. We used the highly efficient TPAV PTE as a positive control for eIF4E binding to an uncapped PTE, as shown previously (56). As a negative control, we used the mutant TPAVm2, which contains mutations (CC to AA) in the bridging domain that inactivate the TPAV PTE and greatly reduce the binding affinity of the uncapped PTE to eIF4E (56). As shown previously, the capped and uncapped TPAV PTE formed a protein-RNA complex, as indicated by the reduced mobility of ^32^P-labeled PTE in the presence of eIF4E (Fig. 7). Also as expected, the uncapped TPAVm2 PTE did not bind to eIF4E except at very high concentrations and most RNA remained unbound, while the capped form of TPAVm2 did bind eIF4E. Some nonspecific binding to any RNA by eIF4E is expected, as it is a low-affinity nonspecific RNA-binding protein (58, 59). Like the wild-type TPAV PTE, both capped and uncapped forms of the MTE bound to eIF4E (Fig. 7). Based on repeated EMSA gels, the approximate dissociation constant (*K_d_*), estimated as the eIF4E concentration at which half of the MTE is shifted, is about 80 nM.

**FIG 8:**
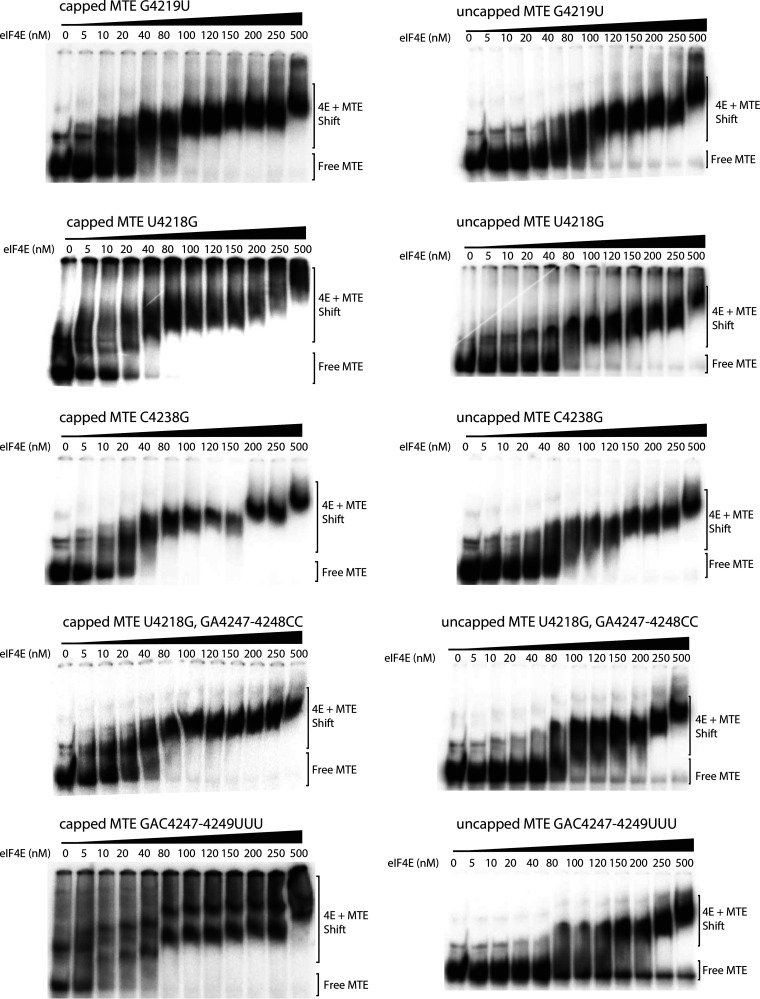
Electrophoretic mobility shift assays (EMSAs) of mutant MTE RNAs with eIF4E. The indicated capped or uncapped ^32^P-labeled transcripts (10 fmol) were incubated with the indicated concentrations of wheat eIF4E prior to electrophoresis on a nondenaturing gel. See the Materials and Methods for details.

We next tested the ability of mutant MTEs to bind eIF4E, in order to determine if eIF4E binding correlates with the translation enhancement function, as was observed previously for the TPAV PTE. Differences in binding affinity can be seen most clearly by the amount of shifted eIF4E at the 100 nM concentration in the gels in Fig. 8. All of the mutants that gave between 50% and 150% translation relative to wild type in Fig. 6 bound eIF4E with roughly similar affinity as the wild-type MTE. Mutant U4218G, GA4247-4248CC gave a more smeared gel shift, possibly due to partial misfolding of the MTE, as detected in the full-length genome context (see below). Mutant C4238G reduced translation 10-fold in the presence of wild-type 5′ UTR (Fig. 5), but its translation activity was restored to 40% of wild type in the presence of a compensating mutation in the 5′ UTR (see below). It bound eIF4E with a similar affinity as wild-type MTE, indicating that the reason for its lack of function was likely due to inability to base pair to the 5′ UTR, and not an inability to bind eIF4E. In contrast to the above mutants, and importantly, MTE mutant GAC4247-4249UUU, which had greatly reduced translation, showed significantly less binding than wild-type MTE at 80 to 200 nM eIF4E and some RNA remained unbound even at the highest eIF4E concentrations (Fig. 8). In this low-resolution assay, there is not precise correlation between the ability of a mutant MTE to bind eIF4E and its ability to stimulate translation in the context of an mRNA. However, it is clear that wild-type MTE binds eIF4E with an affinity similar to that of the previously characterized TPAV PTE, and with much higher affinity than either the negative-control RNA (TPAVm2 PTE), or the mutant MTE that was inactive in translation (GAC4247-4249UUU). Thus, binding of eIF4E to the MTE likely plays a role in MTE-mediated cap-independent translation, as it does for PTE-mediated cap-independent translation (53, 57).

### Secondary structure of the MCMV 5′ UTR.

**FIG 9:**
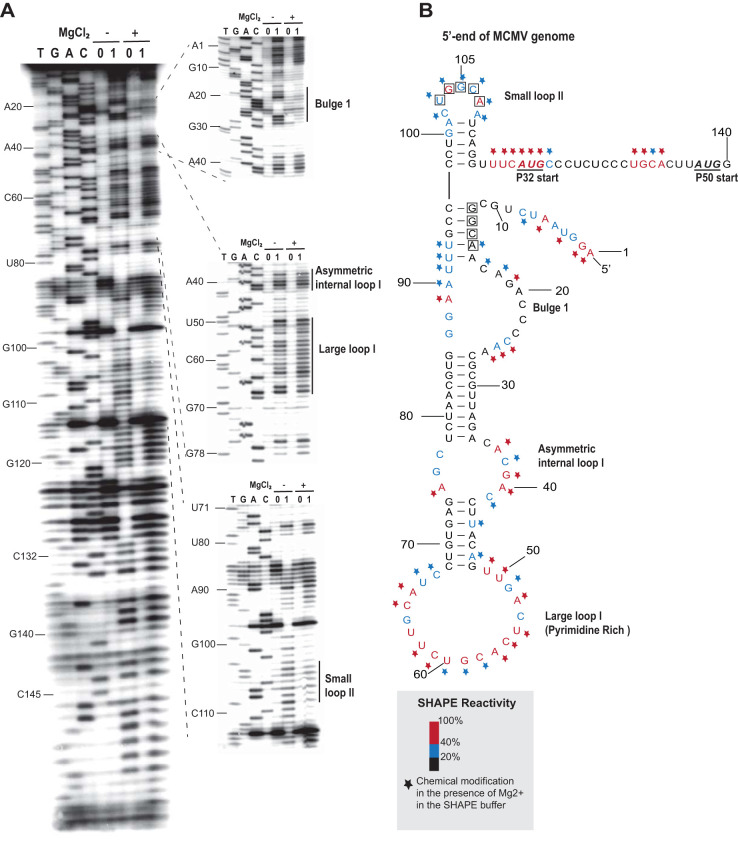
SHAPE analysis of the MCMV 5′ UTR. (A) SHAPE probing gel of the 5′ end of MCMV RNA (nt 1 to 140). RNA was treated with either 60 mM benzoyl cyanide (1) in dimethyl sulfoxide (DMSO) or DMSO only (0) in the presence (+) or absence (−) of magnesium in SHAPE buffer. The sequencing ladders (lanes TGAC) were generated by dideoxy sequencing of RNA with the same 5′-labeled primer used in the modification lanes. Zoom-in sections (gels at right) were obtained in a different gel by allowing electrophoresis to run longer. (B) Superposition of the probe activities on the best-fitting predicted secondary structure of the 5′ UTR. SHAPE activity levels are indicated by color-coded bases, as defined below the structure. Nucleotides inside the squares are tracts that have potential to base pair with loop I of the MTE (Fig. 3). Modification levels of nucleotides in the presence of magnesium are indicated by stars following the reactivity color-coding scheme. AUG start codons are underlined.

Most plant viral 3′ CITEs that have been studied interact with the 5′ end of the viral genomic RNA and subgenomic mRNA via long-distance base pairing of the 3′ CITE to the 5′ UTR, presumably to deliver initiation factors to the 5′ end, where they recruit the ribosomal 40S subunit to the RNA (35, 52, 60–62). Thus, we sought to determine if the same interaction occurs in MCMV RNA. Initial *in silico* analysis of the 5′-end structure of MCMV revealed two sites (GGCA_12–15_ or UGGCA_103–107_) that could potentially base pair with the MTE sequence UGCCA_4236–4240_ in loop 1. An RNA transcript containing the 117-nt 5′ UTR followed by the first 23 nt of the coding region, including the P32 (AUG_118–120_) and P50 (AUG_137–139_) ORF start codons, was subjected to SHAPE probing to determine which region was most likely to be available (single stranded) to interact with the MTE (Fig. 9A). The 5′ UTR was found to consist of a large stem-loop with several large bulges, followed by a short stable stem-loop terminating 5 nt upstream of the start codon (Fig. 9B). The first potential MTE-interacting sequence (GGCA_12–15_) is buried in a stem helix, while the UGGCA_103–107_ is in a favorable loop (Fig. 9B). This led us to suspect that UGGCA_103–107_ is the potential base pairing sequence that interacts with MTE side loop 1.

### Long distance base pairing of the MTE to the 5′ UTR.

**FIG 10:**
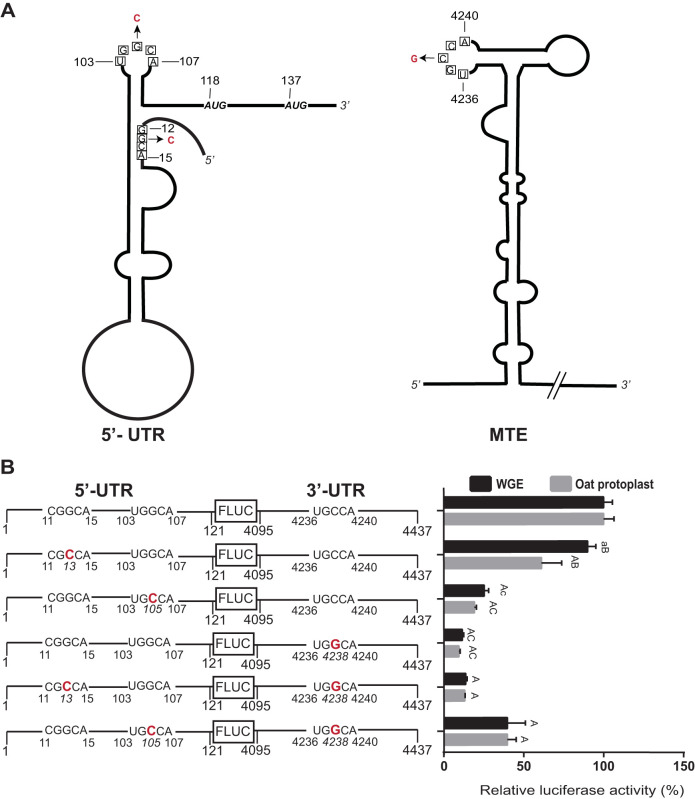
Long-distance interaction between the MCMV 5′ UTR and the MTE. (A) Wire diagrams of the 5′ UTR and the MTE showing tracts (boxed bases) capable of base pairing between the 5′ UTR and MTE. Mutations introduced to disrupt and restore potential long-distance base pairing are indicated in red. AUG start codons are shown at positions 118 and 137. (B) Effects of mutations (enlarged, red letters) on translation of MlucM. Relative luciferase translation activities of the indicated uncapped transcripts in WGE and oat protoplasts are shown as percentages of relative light units relative to MlucM wild type (100%). Data are average percentages (±SD) from 4 independent experiments (for each construct, WGE: *n* = 16; oat protoplasts: *n* = 13). Two-way ANOVA multiple comparison was used to analyze the significance of each set of samples against MlucM_WT_: A, *P* < 0.0001; a, *P* < 0.005. Two-way ANOVA-Dunnett’s multiple-comparison test was performed to compare statistical differences among double mutants and single mutants. Mutants compared with MlucM_G13C/C4238G_ were designated B if *P* < 0.0001 or b if *P* < 0.005. Mutants compared with MlucM_G105C/G4238G_ were designated C if *P* < 0.0001 or c if *P* < 0.005. ANOVA multiple comparisons were performed in the context of each sample collection method (i.e., WGE or oat protoplast).

We next defined functionally which (if any) of the above candidate sequences base pairs to the MTE. Mutations were introduced in the XGCCA regions in the 5′ UTR (nt 11 to 15 or 103 to 107) and the MTE UGCCA_4236–4240_ region (Fig. 10A). Mutation of G_13_ to C caused only a small decrease in luciferase activity in WGE and in oat protoplast translation systems (Fig. 10B). In contrast, mutation of G_105_ to C reduced luciferase activity by ∼75% in WGE and protoplasts. Even more extreme, the C4238G mutation of the middle base in the MTE UGCCA_4236–4240_ tract decreased luciferase activity by 80% to 90% (Fig. 10B, see also Fig. 5C). These mutations were then combined to restore any long-distance base pairing that may have been disrupted. The MlucM_G13C/C4238G_ double mutant, which would restore long-distance base pairing to the 5′-proximal complementary sequence in the 5′ UTR, yielded the same low translation activity as C4238G alone. In contrast, double mutant MlucM_G105C/C4238G_, which is predicted to restore long-distance base pairing of the MTE to the 5′ distal complementary sequence in the 5′ UTR, yielded a 2-fold increase in translation activity compared to MlucM_G105C_ and a 3- to 4-fold increase in translation relative to the more deleterious C4238G single mutation (Fig. 10B). While the compensating mutations did not fully restore a wild-type level of translation, the fact that the double mutant MlucM_G105C/C4238G_ but not MlucM_G13C/C4238G_ translated significantly more efficiently than MlucM_C4238G_ supports base pairing between 5′ UTR nt 103 to 107 and MTE nt 4236 to 4240 as a requirement for efficient cap-independent translation.

**FIG 11:**
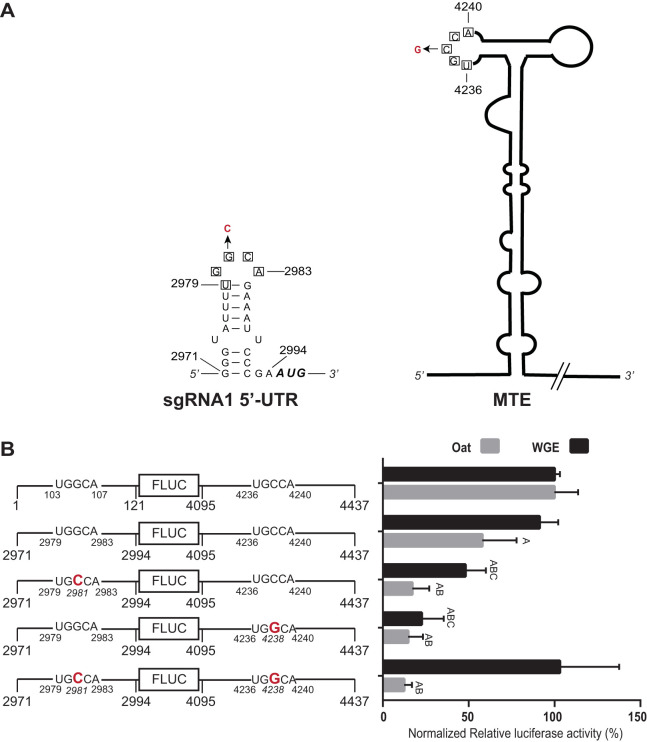
Long-distance interaction between the sgRNA1 5′ UTR and MTE. (A) Wire diagrams of sgRNA1 5′ UTR and MTE showing tracts (boxed bases) capable of base pairing between 5′ UTR and MTE. Mutations introduced to disrupt and restore potential long-distance base pairing are indicated in red. The first start codon is shown at nt 2995. (B) Translation of MlucM (top row) or Msg1lucM (remaining rows), with mutations shown in enlarged red text. Relative luciferase translation activities of the indicated uncapped transcripts in WGE and oat protoplasts are shown as percentages of relative light units relative to MlucM wild type (100%). Data are average percentages (±SD) from 4 independent experiments (for each construct, WGE: *n* = 12; oat protoplasts: *n* = 16). One-way ANOVA multiple-comparison was used to analyze the significance of each set of samples against MlucM_WT_: A, *P* < 0.0001. One-way ANOVA-Dunnett’s multiple-comparison test was performed to compare statistical differences among double mutants and single mutants. Mutants compared with Msg1lucM were designated B if *P* < 0.0001. Mutants compared with Msg1lucM_G2981C/G4238G_ were designated C if *P* < 0.0001. Mutations in UTRs are indicated in boldface red letters.

The MTE should also base pair to the 5′ UTR of sgRNA1, to allow translation of the viral coat and movement proteins. Indeed, we identified a sequence, UGGCA_2979–2983_ in the short 25-nt 5′ UTR of sgRNA1 which matches the UGGCA_103–107_ that base pairs to the MTE. This sequence is predicted to be in the terminal loop of the stem-loop that occupies the 5′ UTR of sgRNA1 (Fig. 11A), which starts at nt 2971 (28). We investigated both the effect of this short 25-nt 5′ UTR on MTE-mediated translation efficiency, and the role of base pairing (if any) between UGGCA_2979–2983_ and UGCCA_4236–4240_ in the MTE. In WGE, the sgRNA1 5′ UTR enabled translation about equally efficiently as the genomic 5′ UTR, while translation was about two-thirds as efficient in oat protoplasts (Fig. 11B), perhaps due to reduced RNA stability conferred by the shorter 5′ UTR. Separate G2981C and C4238G mutations in the sgRNA1 5′ UTR and the MTE, respectively, reduced translation significantly in both WGE and oat protoplasts. The double mutant, designed to restore predicted base pairing by means of G2981C and C4238G in the same construct, gave surprising results. In WGE, as predicted, the double mutant fully restored translation to wild-type levels. However, in oat protoplasts, the same mRNA was as nonfunctional as those containing single G2981C and C4238G mutations, showing no restoration of translation whatsoever. Because WGE is a high-fidelity translation system that measures only translation, independent of the complicated environment of the cell, we conclude that the long-distance base pairing is necessary for efficient cap-independent translation. We speculate that the G2981C mutation altered the RNA in such a way as to make it highly unstable in cells or able to fortuitously interact with cellular components that preclude translation, and which are absent in WGE.

### Effects of mutations on translation of full-length MCMV genomic RNA.

**FIG 12:**
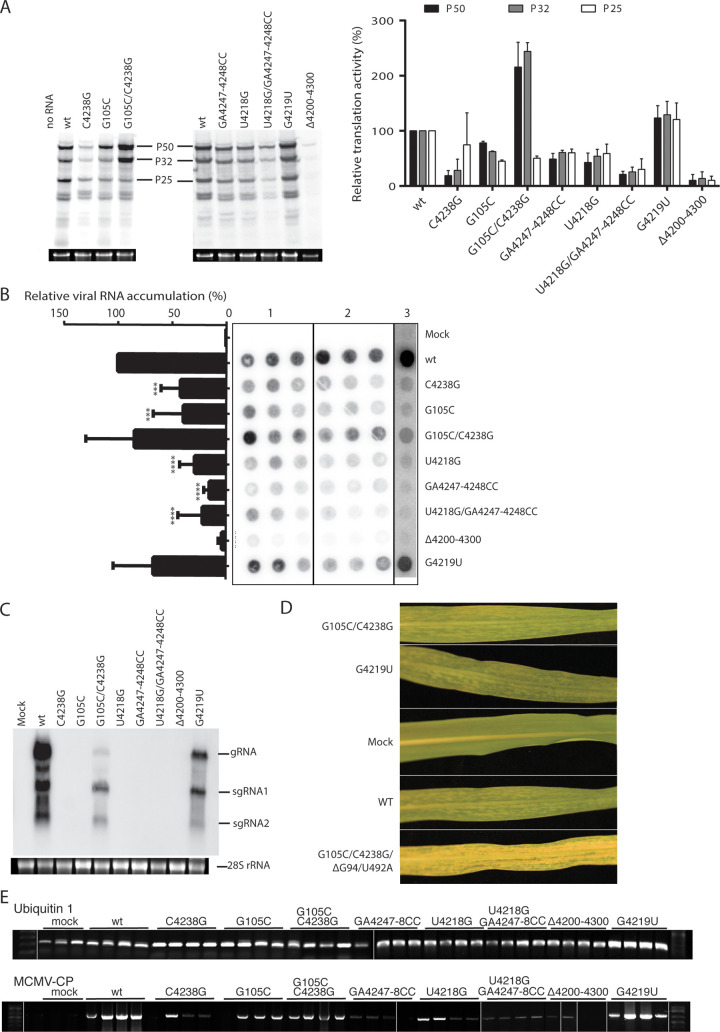
Effects of mutations on translation and replication of the full-length MCMV genome. (A) Top: ^35^S-met-labeled translation products from full-length MCM41 transcript in WGE. Mobilities of P50, P32, and P25 protein products are indicated. Bottom: agarose gels showing *in vitro* transcripts used as mRNA in each lane above. Graph: relative protein levels quantified using ImageQuant from two independent experiments. (B) Dot blots of transfection assays in BMS protoplasts. At 24 hpi, total RNA was extracted and vacuum filtered through a nylon membrane (see the Materials and Methods). Blots were probed with ^32^P-labeled antisense transcript complementary to nt 3811– to 4356 of MCMV genomic RNA, quantified by phosphorimagery, and normalized to that obtained with wild-type MCM41 infection. One-way ANOVA was used to analyze the significance of each set of samples. Three or four asterisks indicate statistical differences with *P* < 0.001 or *P* < 0.0001, respectively. Shown are blots from two experiments performed in triplicate, and one experiment with a single sample for each mutant. Mean relative spot intensity (viral RNA accumulation) for the three independent experiments was plotted in the bar graph on the left side of the panel. (C) Northern blot hybridization of total RNA extracted from maize (B73) plants 14 dpi with the indicated mutants of MCM41. Mobilities of genomic RNA (gRNA), subgenomic RNAs 1 and 2 (sgRNA1 and sgRNA2) are indicated. (D) Symptoms at 14 dpi in systemically infected leaves from plants inoculated with the indicated mutants. The bottom leaf indicates the severe symptoms observed in plants infected with MCM41_G105C/C4238_ in which two spontaneous mutations, ΔG94 and U492A, appeared. (E) Composite image of gels showing RT-PCR of plants inoculated with MCMV mutants. Total RNA isolated from the 3rd leaf (8 dpi) was divided into two fractions, where one underwent RT-PCR to amplify ubiquitin1 mRNA (top) as an internal control, while the other was RT-PCR amplified for the MCMV CP ORF (bottom). Overall RT-PCR screenings are summarized in Table 1.

To determine the effects of mutations that affect translation in the natural context of genomic RNA, selected mutations from the luciferase experiments were introduced into the MCMV infectious clone pMCM41. First, uncapped, full-length genomic RNA transcripts from pMCM41 mutants were translated in WGE, and the predominant ^35^S-met-labeled viral protein products (P50, P32, and P25) were observed. In agreement with the luciferase reporter constructs, mutants MCM41_C4238G_ and MCM41_G105C_, which disrupt the long-distance base pairing between MTE and 5′ UTR, yielded less viral protein than wild type (Fig. 12A). In contrast, the double mutant MCM41_G105C/C4238G_, which restores the long-distance base pairing, translated more efficiently than wild-type RNA for the P32 and P50 proteins. A 25-kDa protein, presumably the viral coat protein (MW 25 kDa), was not expected to be translated much from genomic RNA as seen in Fig. 1B, but it appeared in this experiment. Its translation remained at about the same reduced level in the double mutant as in the single mutants. MCM41 mutants G4219U, U4218G, and Δ4200–4300 translated similarly to the luciferase constructs, relative to wild type (WT). However, the mutant designed to form a GGG:CCC pseudoknot in the MTE, MCM41_U4218G/GA4247-4248CC_, showed a substantial decrease in translation, in contrast to the same mutation in the luciferase reporter, in which translation was increased by 50% (Fig. 6F).

To determine if the mutants that translated poorly in the full-length genome context did so due to gross misfolding of the MTE, we performed SHAPE probing in the context of the MCMV genome (Fig. S2 and S3). For comparison, wild-type MTE folded the same as in Fig. 4. Mutant C4238G, which disrupted long-distance base pairing with 5′UTR, maintained near-wild type MTE secondary structure, where the only difference was that the SHAPE reactivity data decreased in the side loop 1 (4235 to 4241). Functional mutants G4219U and U4218G were also similar in structure to WT, with the exception that in U4218G, the purine-rich bulge was less modified in the absence and presence of magnesium. Interestingly, secondary structures of the MTE mutants designed to have a strong pseudoknot, GA4247-4248CC and U4218G/GA4247-4248CC were changed radically, with either an increased SL-I stem at the expense of the pseudoknot, or formation of an unbranched, multiple-bulged stem-loop structure. Both mutants changed the wild-type MTE structure in such way that forced the reverse transcriptase to stop around nucleotides 4228 to 4241 (∼SL-I), even in the absence of SHAPE chemicals. These SHAPE results may explain the difference in function of this mutant between reporter assay (functional) and viral genomic context (nonfunctional).

### Replication of mutant MCMV RNA.

To test the effects of mutations on viral RNA replication and accumulation, maize protoplasts from a Black Mexican Sweet (BMS) cell culture were transfected with full-length mutant MCM41 transcripts. Unfortunately, in BMS protoplast preparations, high levels of RNase obscured detection of distinct viral RNAs in Northern blot hybridization, so the level of viral RNA was quantified by simple dot blot hybridization. Single mutants MCM41_C4238G_ and MCM41_G105C_, which had disrupted long distance base-pairing, yielded about 35 to 40% as much viral RNA replication product as wild type, while the double mutant MCM41_G105C/C4238G_, yielded about twice as much viral RNA as the single disruptive mutants (Fig. 12B). On the other hand, the mutant designed to form a GGG:CCC pseudoknot, MCM41_U4218G/GA4247-4248CC_, yielded 80% less viral RNA than wild type, whereas replication of MCM41_G4219U_ was not significantly less than wild type. Finally, MCM41_Δ4200–4300_ produced virtually no viral RNA. Overall, efficiently translating mutants replicated at near-wild type levels, while mutants with reduced translation in the full-length context accumulated much lower levels of viral RNA, as expected.

We next attempted to validate the observations in protoplasts by inoculating maize plants with pMCM41 mutant transcripts (average of 36 individual inoculations per mutant). Maize B73 plants inoculated with wild-type MCM41 transcript began to exhibit chlorotic mottling symptoms between 8 and 10 days postinfection (dpi), while sweet corn (Golden Bantam) plants exhibited symptoms around 6 to 9 dpi. Viral RNA was detected via reverse transcriptase PCR (RT-PCR) in inoculated leaves of all plants at 8 dpi, including those that never showed chlorotic mottling. At 14 dpi, samples from the newest systemic leaves were subjected to Northern blot hybridization with a probe complementary to the 3′ end of the MCMV genome (Fig. 12C). Following inoculation of both B73 and Golden Bantam maize plants, only three of the nine MCMV mutants tested elicited symptoms (chlorotic mottling). MCMV RNA was never detected in asymptomatic plants via Northern blot hybridization. Viral RNA from samples displaying positive Northern blot signals was subjected to Sanger sequencing for verification of introduced mutations (Table 1). The few single mutants (C4238G, G105C, and U4218G) that showed symptoms at 14 dpi had reverted to wild type MCM41 (Table 1). MCM41_G4219U_ had a similar infectivity to wild type, but in about half of the infected plants the virus reverted to wild type (Table 1). Moreover, the MCM41_G4219U_ that did not revert to wild type accumulated less RNA (Fig. 12C), suggesting that although the G4219U mutation is tolerated, the wild-type sequence is more competitive. MCM41_G105C/C4238G_ retained its introduced mutations but the infectivity (Table 1) and RNA accumulation (Fig. 12C) was reduced relative to wild type.

**TABLE 1:** Summary of infections in whole plants by wild-type virus versus mutant constructs of MCMV

Construct	Maize genotype	RT-PCR^*a*^	Northern blotting^*c*^	Systemic symptoms^*d*^	% Infection efficiency^*e*^	Virus recovered in systemic infection
Mock	B73	−	−	NA	0 (0/15)	NA^*f*^
	Sweet corn^*g*^	−	−	NA	0 (0/13)	NA
MCM41-WT	B73	+^*b*^	+	Normal	89 (17/19)	MCMV-WT
	Sweet corn	+	+	Normal	88 (21/24)	MCMV-WT
MCM41-C4238G	B73	±	±	NA; delayed, mild	7 (1/15)	Reverted to MCMV-WT
	Sweet corn	±	±	NA; delayed, mild	7 (1/15)	Reverted to MCMV-WT
MCM41-G105C	B73	±	−	NA	0 (0/15)	NA
	Sweet corn	±	±	Delayed, normal	18 (2/11)	Reverted to MCMV-WT
MCM41-G105C, C4238G	B73	+	+	NA; delayed, mild	20 (3/15)	MCM41 G105C, C4238G
	Sweet corn	+	−	Delayed, mild and severe	27 (3/11)	MCM41 G105C, C4238G; MCM41 G105C, C4238G + T422A, ΔG94
MCM41-GA4247-4248CC	B73	±	−	NA	0 (0/17)	NA
	Sweet corn	±	−	NA	0 (0/15)	NA
MCM41-U4218G	B73	±	±	NA, normal	12 (2/17)	Reverted to MCMV-WT
	Sweet corn	±	±	Delayed, normal	7 (1/15)	Reverted to MCMV-WT
MCM41-U4218G, GA4247-4248CC	B73	±	−	NA	0 (0/19)	NA
	Sweet corn	±	−	NA	0 (0/19)	NA
MCM41-Δ4200-4300	B73	±	−	NA	0 (0/17)	NA
	Sweet corn	±	−	NA	0 (0/15)	N/A
MCM41-G4219U	B73	+	+	Normal	88 (15/17)	MCM41-G4219U; Reverted to MCMV-WT [7/15]
	Sweet corn	+	+	Normal	74 (14/19)	MCM41-G4219U; Reverted to MCMV-WT [8/14]

a MCMV coat protein gene detected by RT-PCR at 8 dpi on the 4th leaf. PCR bands detected in plants with no symptoms were faint.

b (+), 70% to 100% of samples tested positive; (−), all samples tested negative; (±), some samples, but ≤60%, tested positive.

c MCMV genomic RNA, sgRNA1, and sgRNA2 from infected plants at 14 dpi were detected by Northern blot hybridization.

d Mottling symptoms were observed on noninoculated new leaves (4–6). Normal, mottling; mild, light mottling; severe, severe yellowing. Variability is between individual plants, not within an individual plant.

e Percentage was calculated by dividing plants showing mottle symptoms/number of plants inoculated.

f NA, not applicable.

g Golden bantam cultivar.

Interestingly, the viral RNA from one sweet corn plant inoculated with the MCM41_G105C/C4238G_ double mutant acquired additional mutations. These spontaneous mutations consisted of deletion of G_94_ in the 5′ UTR and a U492A point mutation in the overlapping P32 and P50 ORFs. This mutation introduced a stop codon in the P32 frame, shortening the protein from 289 aa to 125 aa, and changed amino acid 119 from valine to glutamic acid in the P50 and P111 (RdRp) proteins. This spontaneous mutant did not induce symptoms until 12 dpi, but after 14 dpi, symptoms were more extreme than wild type, giving nearly translucent leaves (Fig. 12D). In summary, the MCMV genome tolerated few mutations, and only those mutations that allowed the most efficient translation replicated in maize plants.

## DISCUSSION

### Identification of the 3′ CITE in the MCMV genome.

Given the severe losses it has caused since 2011 in mixed infection with the potyvirus SCMV in East Africa (7, 63, 64), China (10, 65, 66), and Ecuador (13), and given the cost of screening seed to ensure absence of this seed-transmitted virus (63, 67, 68), MCMV is almost certainly the most economically important virus in the >76-member *Tombusviridae* family (23).Thus, it is imperative to understand the life cycle of MCMV, including translation, to identify molecular targets for genetic approaches to control this virus. All tombusvirids that have been studied contain a 3′ CITE (35, 40, 60, 69, 70), yet these are not predictable because the class of 3′ CITE a tombusvirid possesses does not always correlate with phylogeny (35, 52). Moreover, the class of 3′ CITE in MCMV RNA was not predictable using ViennaFold or MFOLD. Therefore, we used experimental approaches to determine that MCMV contains a 3′ CITE in the PTE class, which we call MTE. Unlike the most efficient PTEs, the MTE lacks a strong pseudoknot and stimulates cap-independent translation less efficiently than most PTEs.

Previous studies of nine PTEs revealed a common secondary RNA structure composed of a branched structure with two side loops (Fig. 5) connected by a pyrimidine-rich bridging domain (52) and a purine-rich bulge (formerly called the G bulge) in the basal stem. A uniformly conserved feature is a guanosine in the purine-rich bulge that can be hypermodified by SHAPE reagents such as benzoyl cyanide in the presence of magnesium (53). Despite this hypermodification, previous evidence also supports a pseudoknot interaction between purine-rich bulge bases and the bridging domain (53). In the case of the MTE and some PTEs, the predicted pseudoknot interaction is tenuous, as the bridging domain is not always pyrimidine-rich; however, a magnesium-dependent hypermodified G in the G-bulge is still present. In the PMV and PEMV2 PTEs, this region is protected from SHAPE reagents by eIF4E and is thus the likely eIF4E-binding site (53). The structural basis of this interaction is unclear, but the strongest PTEs have a relatively strong pseudoknot base pairing (GGG:CCC), whereas PTEs that have weak or no Watson-Crick base pairs are generally less stimulatory of translation (Fig. 5). But given that strong pseudoknot base pairing does not guarantee an efficient PTE (e.g., PEMV2 and HRSV), we conclude that, for PTEs, a strong pseudoknot appears to be necessary but not sufficient for efficient cap-independent translation. With regard to the MTE, a mutant designed to have a strong(er) pseudoknot yielded 50% more translation product than the wild-type MTE in the luciferase reporter context (MlucM_U4218G/GA4247-4248CC_, Fig. 6F), but these same mutations reduced translation, and thus replication, when in the context of the MCMV full-length clone (Fig. 12), owing to misfolding. Other mutants designed to have pseudoknots also reduced translation because of misfolding (Fig. S3).

### MTE binding to eIF4E.

Binding of wild-type MTE to eIF4E is fairly tight (apparent *Kd* ∼80 nM), but some MTE mutants that did not confer efficient translation still bound eIF4E with high affinity, while others did not. Importantly, all functional mutants bound eIF4E with high affinity (approximate *Kd* ≤100 nM), consistent with a requirement for this factor. eIF4E binding by nonfunctional mutants may be due to different folding of the RNA in the short, isolated sequence context used in EMSA, or these mutants may have lost the ability to bind other translation components required in addition to eIF4E. eIF4E facilitates translation while bound to its partner eIF4G, forming the heterodimer eIF4F. It is possible that the eIF4G component may affect binding affinity to the MTE either by altering eIF4E conformation or by binding directly to the MTE as well. While the PTEs bind directly to eIF4E, barley yellow dwarf virus-like 3' CITEs (BTEs) bind directly to eIF4G, and these and other types of 3′ CITE all bind with higher affinity to eIF4F than to eIF4E or eIF4G alone (40–43, 57). Thus, future experiments are necessary to determine how eIF4F and possibly other proteins bind to the MTE to determine the specific RNA-protein interactions that facilitate cap-independent translation. Also of future interest would be identification of eIF4E mutants that lose affinity for the MTE but retain cap-binding activity. These would be candidates for recessive resistance genes (71, 72).

### Long-distance kissing stem-loop interactions.

A sequence in the 5′ UTR complementary to a loop in the 3′ CITE required for efficient translation has been found in most 3′-CITE-containing viral genomes (35). This presumably facilitates initiation factor-mediated recruitment of the ribosomal subunit to the 5′ end of the RNA (35, 52, 60–62, 73). The sequence of the MTE long-distance kissing stem-loop interactions, between either the genomic 5′ UTR or the subgenomic RNA 5′ UTR and the MTE is UGGCA:UGCCA. This sequence fits the consensus found in many tombusvirids: YGGCA:UGCCR (35, 52), supporting our experimental evidence (Fig. 10 and 11). Why this particular sequence pair has been conserved is unknown.

Recent papers have shed light on the puzzle of how such short complementary sequences can “find each other” across the length of the genome. Lai et al. (74) provided computational and experimental evidence using FRET that mRNAs and long noncoding RNAs naturally fold in a way to bring the 5′ and 3′ ends into close proximity, unless they are very low in complexity and guanosine residues. This folding would obviously facilitate the base pairing between 5′ and 3′ UTRs required for 3′ CITE function. Also, in the case of the BTE (but not I-shaped 3′ CITEs), the long-distance interaction may be enhanced by translation initiation factor eIF3, which is reported to bind both the BTE and the 5′ UTR of BYDV (75).

We have not ruled out the possibility that other sequences within the MCMV genome in addition to the MTE may stimulate cap-independent translation initiation. A reporter construct containing in its 3′ UTR the 3′ end of the 3′-proximal ORF (CP ORF), as well as the entire 3′ UTR, stimulated translation more than the 3′ UTR alone (Fig. 3). It is distinct from the MTE, as the 75-nt sequence between the CP ORF stop codon and the MTE is unnecessary for cap-independent translation. Moreover, deletions in the viral genome (Fig. 1) and in reporter constructs (Fig. 3, Fig. 4) reveal that the CP ORF is insufficient for translation stimulation *in vitro* or *in vivo*.

It is also possible that sequence in the 5′ end of the coding region (ORFs P32 and P50), in addition to the UGGCA_103–107_ in the 5′ UTR, could participate in the long-distance base pairing. For example, Simon’s group reported that a stem-loop in the 5′ end of the 5′-proximal ORF base pairs to a region adjacent to the PTE in PEMV2 RNA (76). For MCMV, possibilities for base pairing of the 5′ end of the coding region to the UGCCA in the MTE include (i) UGGCG_138–142_, which includes two bases of the P50 ORF start codon and requires a G-U pair, (ii) UGGC_179–182_, and (iii) UGGC_287–290_. However, none of these would form as stable a base pairing to UGGCA as the UGGCA_103–107_ sequence we identified as required for base pairing to the MTE. Further, SHAPE probing indicates that the three middle bases of the UGGCG_138–142_ motif are base-paired and thus unlikely to be accessible for the long-distance interaction. These sequences may also be too far from the 5′ terminus to allow efficient delivery of translation factors for entry of the ribosomal 40S subunit at the 5′ end. In contrast, the PEMV2 sequence in the coding region is just 71 to 76 nt from the 5′ end, and in a clearly identifiable loop of a strong stem-loop (76).

The translation of CP (P25) from full-length MCM41 RNA observed in Fig. 12A was unexpected, as very little CP was translated in the experiment shown in Fig. 1B. This difference may be due to variation in initiation factor or nuclease levels among batches of WGE used, as the experiments were performed in the Miller (Fig. 12A) and Scheets (Fig. 1B) labs. It is possible that nucleolytic degradation in WGE generates small amounts of sgRNA1-sized RNA available for translation. In fact, in WGE, the noncoding sgRNA2 is indeed generated by exonucleolytic degradation of all but the 3′ UTR of genomic RNA (our unpublished data and reference 77). Alternatively, internal ribosome entry is possible, as Simon’s lab has reported that another tombusvirid, TCV, harbors an internal ribosome entry site (IRES) to allow direct translation of CP from genomic RNA (as well as from sgRNA) (78). However, the TCV IRES region is a tract of unstructured RNA, and we found no similar sequence upstream of the MCMV CP start codon.

The MCM41_C105G/G4238C_ transcript, which translates more efficiently than wild type in WGE, replicates indistinguishably from wild-type MCM41 in protoplasts, but accumulates to much lower levels in whole plants. This is to be expected because the G4238C mutation in the MTE would prevent base pairing to the 5′ end of sgRNA1, which remains a wild -type sequence. Thus, we predict translation of the CP and movement proteins is reduced in this construct. CP and movement proteins are not necessary for replication of other tombusvirids in protoplasts but would, of course, be necessary for the virus to accumulate in plants, thus explaining the difference in accumulation in protoplasts versus plants (Fig. 12B and C).

### Translation of sgRNA1.

Comparison of translation efficiencies of genomic (MlucM) and subgenomic RNA (Msg1lucM) reporter constructs revealed no striking difference in translation efficiency conferred by the 5′ UTR (Fig. 11). This is interesting, because the secondary structure and length of the 5′ UTR is much less in the sgRNA1 5′ UTR (24 nt, Δ*G* = −6.6 kcal/mol) compared to that of genomic RNA 5′ UTR (117 nt, Δ*G* = −21.2 kcal/mol). We expected the sgRNA1 5′ UTR to confer superior translation efficiency because it should provide less resistance to ribosome scanning. Perhaps in infected cells, in the presence of the abundant sgRNA2, which contains the MTE, the sgRNA1 5′ UTR could outcompete the genomic RNA 5′ UTR for limiting eIF4E, as discussed below.

In addition to stimulating translation of genomic RNA and sgRNA1 in *cis*, the MTE may regulate translation initiation in *trans*. Like certain other tombusvirids, such as red clover necrotic mosaic virus (RCNMV) (79, 80), tobacco necrosis virus-D (81), and the flaviviruses (82), MCMV generates a noncoding sgRNA (sgRNA2) corresponding to most of the 3′ UTR (28). These RNAs are generated by exonucleolytic degradation of the larger viral RNAs until the exonuclease reaches a blocking structure called xrRNA, at which point it stops, leaving the sgRNA, the 337-nt sgRNA2 in the case of MCMV, intact (77). Because this abundant RNA contains the MTE, we propose that it regulates viral and host translation by binding and sequestering the eIF4E subunit of eIF4F (83). sgRNA2 of BYDV (related to the *Tombusviridae*) binds the eIF4G subunit of eIF4F and, as a result, inhibits translation of BYDV genomic RNA (gRNA) while favoring translation of BYDV sgRNA1, which, like MCMV sgRNA1, codes for movement and coat proteins. This would free genomic RNA of ribosomes, making it available for replication, encapsidation, and cell-to-cell movement. The selective inhibition of BYDV gRNA is due to its highly structured 5′ UTR, which likely increases dependence on eIF4F relative to the unstructured 5′ UTR of sgRNA1 (84). The same regulation may occur on MCMV RNA. Thus, the MTE may play an essential role in MCMV infection by acting in *trans*. The role of sgRNA2 may be relevant to flaviviruses, which also produce noncoding sgRNAs from the 3′ UTRs that bind a variety of host proteins (82, 85–87), and which can affect translation (88).

### Toward host resistance to MCMV.

As mentioned above, understanding translation may lead to strategies for resistance. The MTE binds eIF4E via what must include different molecular contacts than the binding of a 5′ m^7^G cap to eIF4E via the cap-binding pocket (89). Thus, it may be possible to identify mutants of eIF4E that lose the ability to bind the MTE but retain a functional cap-binding pocket to allow translation of host (capped) mRNAs. In melon, such a resistance mechanism has been identified against melon necrotic spot virus (MNSV). A point mutation in melon eIF4E confers recessive resistance to most strains of MNSV, while having no negative effect on melon agronomic performance (90, 91). This mutation was shown to preclude translation of MNSV RNA (thus blocking infection) by preventing efficient binding of eIF4E to the MNSV 3′ CITE (an I-shaped structure) (92). In the case of MCMV, in addition to traditional screening of maize genotypes for MCMV resistance (which has achieved limited success), directed studies could either identify natural eIF4E alleles or guide construction of engineered eIF4E mutants that bind poorly to the MTE while still binding capped host mRNAs with high affinity, in order to achieve durable, recessive resistance to MCMV.

## MATERIALS AND METHODS

### Plasmids.

The full-length infectious clone of the MCMV genome (pMCM41) and pMCM721 were described previously (29). For psgRNA1 construction, template DNA pMCM41 was used to amplify MCMV nt 2972 to 4437 using Vent DNA polymerase, and oligonucleotides 3′SmaI (5′-agcaagcttcccGGGCCGGAAGAG [29]) and sgRNA1 (5′-GGTATTTTGGCAGAAATTCC) that were phosphorylated with T4 polynucleotide kinase. The vector pT7E19 (93) was digested with SacI followed by mung bean nuclease digestion. Vector and insert were digested with HindIII, phenol-chloroform extracted, and precipitated prior to ligation with T4 DNA ligase. DNA was added to competent E. coli DH5α cells, selected on ampicillin/XGal plates, and screened by restriction digests and sequencing. Transcripts made from psgRNA1 linearized with SmaI contain MCMV nt 2971 to 4437, the complete sgRNA1. All enzymes were from New England BioLabs. MlucM was constructed using a Gibson Assembly kit (New England BioLabs) such that a firefly luciferase (luc2, Promega) reporter gene was flanked by the 5′ and 3′ UTRs of MCMV (Fig. 1). A Q5 site-directed mutagenesis kit (New England BioLabs) with custom forward and reverse primers (Table S-T1 in the supplemental materials) was used to generate the deletions (Fig. 2) and mutants (Fig. 6, 10 and 11) on the UTRs of the luciferase constructs. Resulting luciferase plasmid constructs were verified by sequencing at the Iowa State University DNA Sequencing Facility.

### *In vitro* transcription.

At Iowa State University (all experiments except that shown in Fig. 1), plasmid DNA templates were linearized by restriction digestion or amplified by PCR to ensure correct template length. The RNAs were synthesized by *in vitro* transcription with T7 polymerase using MEGAscript (for uncapped RNAs) or mMESSAGE mMACHINE (for capped RNAs) kits (Ambion). RNAs used as probes for uncapped EMSAs were generated using MEGAshortscript kit (Ambion). RNA transcripts were purified according to the manufacturer’s instructions and the RNA clean and concentrator kit (ZymoResearch) was used for nonradioactive RNA preparation. RNase-free Bio-Spin columns P-30 (Bio-Rad) were used for radiolabeled RNA. RNA integrity was verified by 0.8% agarose gel electrophoresis. RNA concentration was determined by spectrophotometry for nonradiolabeled RNA. Radiolabeled RNA concentration was calculated by measuring the amount of incorporated radioisotope using a scintillation counter. At Oklahoma State University (Fig. 1), full-length (nt 1 to 4437) or 3′-truncated (nt 1 to 4195) genomic RNA was synthesized with T7 RNA polymerase (New England BioLabs) and either pMCM41 or pMCM721 linearized with Sma I or SpeI following the manufacturer’s protocols for uncapped or capped RNA synthesis. Unincorporated NTPs were removed by three rounds of ammonium acetate/ethanol precipitation and resuspension in nuclease-free water. pMCM721 contains a G residue between the T7 promoter and MCMV sequence, allowing synthesis of capped or uncapped RNAs 1 nt longer than WT (29). The 3K MCMV RNA transcript templates were made by PCR using primers p9KO2 (CAGAAATTCCCGAgTGTC, nt 2982 to 2999) and M13 forward (−20) with both plasmid templates. These oligonucleotides were synthesized by the Oklahoma State University Core Facility.

### *In vitro* translation.

*In vitro* translation reactions were performed in WGE (Promega) as described (94). Nonsaturating amounts of RNAs (30 fmol) were translated in WGE in a total volume of 12.5 μl with amino acid mixture or [^35^S]methionine amino acid mixture (*C_t_* = 3.06 μCi/0.14 MBq, 5.8 Ci/mmol; Perkin Elmer), 93 mM potassium acetate, and 2.1 mM MgCl_2_ based on the manufacturer’s instructions. Translation reaction mixtures were incubated at room temperature (25°C) for 30 min. Translation products were separated by electrophoresis on a NuPAGE 4–12% Bis-Tris gel (Invitrogen), detected with Pharox FX plus Molecular Imager and quantified by Quantity One one-dimensional analysis software (Bio-Rad). For luciferase reactions, 10 μl of the translation reaction product was mixed into 50 μl of Luciferase assay reagent (Promega) and measured immediately on a GloMaxTM20/20 luminometer (Promega). Statistical and data analysis were performed using GraphPad Prism software.

### Translation in protoplasts.

Uncapped MlucM (10 pmol) or its derived mutants were coelectroporated into ∼2 × 10^6^ oat (*Avena sativa* cv. Stout) protoplasts along with capped mRNA encoding *Renilla* luciferase (1 pmol) (95) as an internal control. Protoplasts were prepared and assays performed as described previously (96). After 4 h of incubation at room temperature, protoplasts were harvested and lysed in Passive Lysis Buffer (Promega). Luciferase activities were measured using the Dual Luciferase Reporter Assay System (Promega) in a GloMax 20/20 luminometer (Promega). To minimize variation among electroporation replicates, firefly luciferase activities were normalized to the *Renilla* luciferase. Background firefly relative light units (RLUs), measured in the absence of added luciferase mRNA, were subtracted from the values obtained with MlucM and its deletions and/or mutant derivatives. Statistical and data analysis were performed using GraphPad Prism software.

### RNA structure probing.

Selective 2′-hydroxyl acylation analyzed by primer extension (SHAPE) was applied to selected UTR fragments of MCMV following the procedure previously described (97) for the gel shown in Fig. 3. In Fig. 6 and supplemental data, SHAPE was conducted in the context of the complete genome instead of using the SHAPE cassette. In brief, 500 ng of RNA was denatured by heating to 95°C and renatured in SHAPE buffer (50 mM HEPES-KOH [pH 7.2], 100 mM KCl, and ± 8 mM MgCl_2_) for 30 min at 30°C. RNA was modified by mixing 1/10 (vol/vol) ratio of renatured RNA with 60 mM benzoyl cyanide (Sigma) in anhydrous dimethyl sulfoxide (DMSO) (Sigma). After 2 min at room temperature, the RNA was mixed with 4-fold excess tRNA and precipitated in 3 volumes of 99% ethanol and 1/10 volume 3 M sodium acetate. Control RNA was treated with the same amount of DMSO without benzoyl cyanide. The primer (3′ UTR: 5′-TACTCCGTTGAGTTCAGAAACC-3′, or 5′ UTR: 5′-CCATAAGTGCAGGGAGAGGG-3′) was 5′-end-labeled with [γ-^32^P]ATP and used for primer extension. Gel electrophoresis conditions and phosphorimager visualizations were performed as described previously (57, 97).

### Expression and purification of wheat eIF4E.

Wheat eIF4E pET22b plasmid clone (98) was obtained from Karen Browning. Plasmid was introduced into E. coli (BL21) cells and eIF4E expression was induced at an optical density at 600 nm (OD_600_) of 0.7, with 0.5 mM IPTG (isopropyl-β-D-thiogalactopyranoside). From here on, the purification procedure of the protein was similar to previous published work (98). In short, eIF4E expression in E. coli cells was induced by incubating in IPTG for 2 h at 37°C with shaking (160 rpm). Cells were harvested by centrifugation at 6,000 × *g* for 15 min at 4°C, and cell pellets were quick frozen before purification. E. coli cells were disrupted by sonication in buffer B-50 (20 mM HEPES-KOH [pH 7.6], 0.1 mM EDTA, 1 mM DTT, 10% glycerol, 50 mM KCl) containing complete protease inhibitor cocktail tablets (Roche). Cell debris was separated from the supernatant by 3 to 5 rounds of centrifugation (16,000 × *g* for 15 min), where for each centrifugation the supernatant was transferred to a clean round-bottom centrifugation tube. Wheat eIF4E protein was purified using m^7^GTP agarose beads as described previously (98). Protein was eluted using buffer B-100 (20 mM HEPES-KOH [pH 7.6], 0.1 mM EDTA, 1 mM DTT, 10% glycerol, 100 mM KCl) plus 20 mM GTP. Protein purity was evaluated in a 4–12% NuPage Bis-Tris gel (Invitrogen). Protein concentration was determined by spectrophotometry and using the Bio-Rad protein assay kit with a BSA protein standard curve.

### Electrophoretic mobility shift assay.

Calculated specific activities of probes were used to determine the molarity of RNA in each purified stock. RNA probes were subjected to 6 M urea-TBE gel electrophoresis to verify the quality of the RNA. RNA was diluted to 10 fmol/10 μl for electrophoretic mobility shift assays (EMSAs). As previously described (56),^32^P-RNA-labeled probes were incubated at the indicated wheat eIF4E protein concentrations in EMSA binding buffer. Protein and RNA probes were incubated in a total volume of 10 μl of 1× EMSA binding buffer (10 mM HEPES [pH 7.5], 20 mM KCl, 1 mM dithiothreitol, 3 mM MgCl_2_), 0.1 μg/μl yeast tRNA, 1 μg/μl bovine serum albumen, 1 unit/μl RNaseOUT recombinant RNase inhibitor (Invitrogen), and 20 mM Tris-HCl/10% glycerol for 25 min in ice. Then, 3 μl of 50% glycerol was added to each reaction. Immediately after, 7 μl of RNA-protein mixture was loaded into a 5% polyacrylamide (acrylamide:*bis*-acrylamide 19:1), Tris-borate/EDTA (TBE) gel, which was run at ∼4°C at 110V for 45 min in 0.5× cold TBE buffer. Gels were dried on Whatman 3MM paper and exposed to a phosphorimager screen overnight. Phosphor screens were scanned in a Bio-Rad PhosphorImager, and radioactivity counts were analyzed using Quantity One software (Bio-Rad). Statistical analysis was performed using GraphPad Prism software.

### Inoculation of Black Mexican Sweet protoplasts.

Protoplasts were isolated from Black Mexican Sweet (BMS) suspension cultures as described previously (28, 29). For each experiment, aliquots of isolated protoplasts (2.0 × 10^6^) were transfected with 5 pmol of pMCM41 and pMCM41 mutant transcript RNA using PEG-1540 (40% PEG, 6 mM CaCl_2_, 5 mM MES, pH 5.6). Protoplasts were diluted slowly in 5 ml of solution M (8.5% mannitol, 5 mM MES, pH 5.6, 6 mM CaCl_2_), followed by incubation at 4°C for 20 min. Protoplasts then were centrifuged at 100 × *g* for 5 min, followed by two washes with solution M. PGM buffer (6% mannitol, 3% sucrose, M5524 MS salts [Sigma], M7150 vitamins [Sigma], 0.005% phytagel) was then added to each protoplast sample, followed by incubation in the dark at room temperature for 24 h. RNA was isolated using Plant RNAeasy kit (Qiagen) following the manufacturer’s instructions. Purified RNA was analyzed on a 0.8% native agarose gel and quantified using a Nanodrop spectrophotometer.

### Plant inoculations.

Maize (B73 and sweet corn varieties) was grown in growth chambers on a 16:8 photoperiod with a temperature setting of 25°C/22°C (day/night). Maize seedlings were inoculated at the three-leaf stage. Plants were dusted with carborundum and inoculated on the third leaf with 10 μg of purified MCMV transcripts in 10 mM sodium acetate, 5 mM calcium chloride, and 0.5% bentonite by stroking three times with a freshly gloved finger. Inoculated leaves were rinsed with water 10 min postinoculation, and rinse water was collected and autoclaved prior to disposal. Total plant RNA was extracted using TRIzol (Invitrogen) from 100 mg of the newest systemic leaves for Northern blot hybridization (14 dpi) and cDNA synthesis. Quality of RNA was evaluated in a 0.8% native agarose gel, and RNA was quantified using a Nanodrop spectrophotometer. RNA extracted from infected plants was subjected to cDNA synthesis and reverse transcriptase PCR (RT-PCR) and sent to the Iowa State University DNA Facility for sequencing to evaluate the presence of introduced mutations.

### Dot blot and Northern blot hybridizations.

RNA isolated from BMS protoplasts had a higher ratio of degraded RNA genomic/subgenomic RNA in Northern blot hybridizations, such that lower molecular weight fragments overpowered the genomic and subgenomic RNAs. However, a trend on the amount of MCMV RNA detected suggested that replication was still occurring in protoplasts; thus, dot blots were used instead. Unfractionated RNA from protoplasts was denatured prior to immobilization on a nylon membrane using a vacuum manifold apparatus as described in Brown et al. 2004 (99). RNA from maize plants was not degraded, so it was subjected to Northern blot hybridization. Total RNA from 100 mg of samples from the newest leaves at 14 dpi was denatured in a formaldehyde/formamide buffer solution by heating at 65°C for 15 min, followed by separation in a 0.8% denaturing agarose gel and transfer to a nylon membrane. Both dot blot and Northern blot nylon membranes were UV cross-linked, followed by prehybridization (50% formamide, 5× SSC, 200 μg/ml polyanetholesulfonic acid, 0.1% SDS, 20 mM sodium phosphate, pH 6.5) at 55°C for 2 h. Membranes were hybridized using ^32^P-labeled probe complementary to MCMV nt 3811 to 4356, which had been transcribed using SP6 RNA polymerase. Washed blots were wrapped in plastic and exposed to phosphor screens. Phosphor screens were scanned in a Bio-Rad PhosphorImager and radioactivity counts were analyzed using Quantity One software (Bio-Rad). Statistical analysis was performed using GraphPad Prism software.

### RT-PCR.

The presence of MCMV virus RNA was evaluated by RT-PCR. Total RNA isolated from inoculated maize plants was extracted from washed 3rd leaves using TRIzol. The concentration and purity of extracted RNA was confirmed using a NanoDrop ND-2000 spectrophotometer and the quality of RNA was evaluated via 0.8% native agarose gel electrophoresis. One microgram of RNA was subjected to cDNA synthesis following the manufacturer’s instructions for Maxima first strand cDNA synthesis kit (Thermo Fisher Scientific). The cDNA was amplified using MCMV-CP primers (R: 5′-TGTGCTCAATGATTTGCCAGCCC; F: 5′-ATGGCGGCAAGTAGCCGGTCT) for 25 cycles, and the products were separated on 1% agarose gels, visualized by SYBR safe DNA stain (Thermo Fisher Scientific) and photographed. Similar to MCMV-CP, maize ubiquitin cDNA expression (F: 5′-TAAGCTGCCGATGTGCCTGCGTCG; R: 5′-CTGAAAGACAGAACATAATGAGCACAG) was analyzed in the same sample set to serve as an endogenous positive control.

## Supplementary Material

Supplemental file 1
