# Supplementary material for: The RNA of Maize Chlorotic Mottle Virus, an Obligatory Component of Maize Lethal Necrosis Disease, Is Translated via a Variant Panicum Mosaic Virus-Like Cap-Independent Translation Element
Source: J Virol. 2020 Oct 27;94(22):e01005-20. doi: 10.1128/JVI.01005-20 (PMC7592216; doi:10.1128/JVI.01005-20)
Supplement: Supplemental file 1 [file JVI.01005-20-s0001.pdf]

Supplemental information

Table S1. Table of primers. List of primer sets used in this publication.

Fig. S1. Secondary structures of known and predicted PTEs. The bases predicted to interact with the 5' UTR are inside black boxes. Base pairs predicted to form pseudoknot between the purine-rich bulge and the connecting bridge are indicated with dash lines.

S2 Fig. SHAPE probing of mutant MTE sequence in MCM41 mutant constructs used for infectivity assays. (A) SHAPE probing gel of MTE in the whole genome context of MCM41 mutants. RNA was modified with either 60 mM benzoyl cyanide (1) in dimethyl sulfoxide (DMSO) or DMSO only (0) in the presence (+) or absence (-) of magnesium in SHAPE buffer. The sequencing ladders (lanes AC) were generated by dideoxy sequencing of RNA with the same 5'-labeled primer used in the modification lanes. (B) Reactivity information was superimposed into the characterized MTE structure. To compare where modifications occurred in the presence of magnesium among all mutants, specific geometric shapes were assigned to each hyper-modified nucleotide. Five-point star: wt, four-point star: C4238G, hexagon: GA4247-4248CC, rhombus: U4218G, triangle: GA4247-4248, U4218G, cross: G4219U. Color of each geometric figure corresponds to reactivity data coloring.

Fig. S3. MTE SHAPE structures of selected mutants. SHAPE data was superimposed into best fitted secondary structures. The base of the long stem on MTE was conserved throughout all the structures, thus only areas with major changes are shown. SHAPE reactivity data color-

coding is the same as previous figure. Major changes in the MTE structure are indicated by the creamy-yellow shading.

Constructs: Deletion or mutation	Forward Primer	Reverse Primer
Δ4200-4300	5'-ACGGTGCGACATGGTAAC-3'	5'-AACTACTAGTATACGATTTAGGC-3'
Δ4095-4164	5'-AAAGTGTTTGGGAGCCTAAATC-3'	5'-CGCGGCTTACAATTTGGACTTTC-3'
Δ4095-4191	5'-CTAGTAGTTTGCGTGATGAC-3'	5'-CGCGGCTTACAATTTGGACTTTC-3'
AC4248-4249GG	5'-GCCAACCGCAGGGTGGGCGTATATAGTAAGCCTTGACC-3'	5'-AGCCGCCGCCCACTCTCC-3'
UGG4218-4220CCC	5'-TGACCATGACCCCAGAGTGGGCG-3'	5'-TCACGCAAACTACTAGTATAC-3'
GA4247-4248CC	5'-GCCAACCGCACCCTGGGCGTATATAGTAAGCCTTGACCC-3'	5'-AGCCGCCGCCCACTCTCC -3'
U4218G	5'-TGACCATGACGGGAGAGTGGG-3'	5'-TCACGCAAACTACTAGTATAC-3'
GAC4247-4249UUU	5'-GCCAACCGCATTTTGGGCGTATATAGTAAGCCTTGACCCAC-3'	5'-AGCCGCCGCCCACTCTCC -3'
UGG4218-4220AAA	5'-TGACCATGACAAAAGAGTGGGCG -3'	5'-TCACGCAAACTACTAGTATAC -3'
G4219U	5'-TGACCATGACTtGAGAGTGGG-3'	5'-TCACGCAAACTACTAGTATAC-3'
A4248U	5'-GCCAACCGCAGTCTGGGCGTATATAGTAAGCCTTGACC-3'	5'-AGCCGCCGCCCACTCTCC-3'
U4218A	5'-TGACCATGACAGGAGAGTGGG-3'	5'-TCACGCAAACTACTAGTATAC-3'
MTE EMSA probe	5'-AATTAATACGCTCACTATAGGGTATACTAGTAGTTTGCGT-3'	5'-TGTATCCAGTTACCATGTCGCACCG-3'
TPAV EMSA probe	5'-taatacgactcactataggGCTCTATCCGAAACTCCAGTG-3'	5'-ACTCTCTACCTTCCGTCCAG-3'
G13C	5'-GTAATCTGCGCCAACAGACCC -3'	5'- CTCCCTATAGTGAGTCGTATTAGTG -3'
G105C	5'-CCCCTGACTGCCAATCAGGTTTC -3'	5'-AAATTCCCACGTTAGAGCTC -3'
C4238G	5'-GCGGCGGCTGgCAACCGCAGA-3'	5'-CCACTCTCCAGTCATGGTCATCACGCAAAC-3'
sg1MlucM	5'-AGAAATTCCCGACGCGCGCATGGAAGACGC -3'	5'-GCCAAAATACCCCCTATAGTGAGTCGTATTAGTGGCTTTACC -3'
G2981C	5'-AGAAATTCCCGACGCGCGCATGGAAGACGC -3'	5'-GGCAAAATACCCCCTATAGTGAGTCGTATTAGTGGCTTTACC -3'
MCMV-coat protein	5'-ATGGCGGCAAGTAGCCGGTCT -3'	5'- TGTGCTCAATGATTTGCCAGCCC -3'
Maize Ubiquitin 1	5’-TAAGCTGCCGATGTGCCTGCGTCG -3'	5’- CTGAAAGACAGAACATAATGAGCACAG -3'
Northen Blot probe	5'-ctgaATTTAGGTGACACTATAGTTCCTAGCATCTACTTGCCCC -3'	5'-GGATCGTGCCCTCAGCTACAATAGCTCTGAA -3'

Supplementary Table S-T1

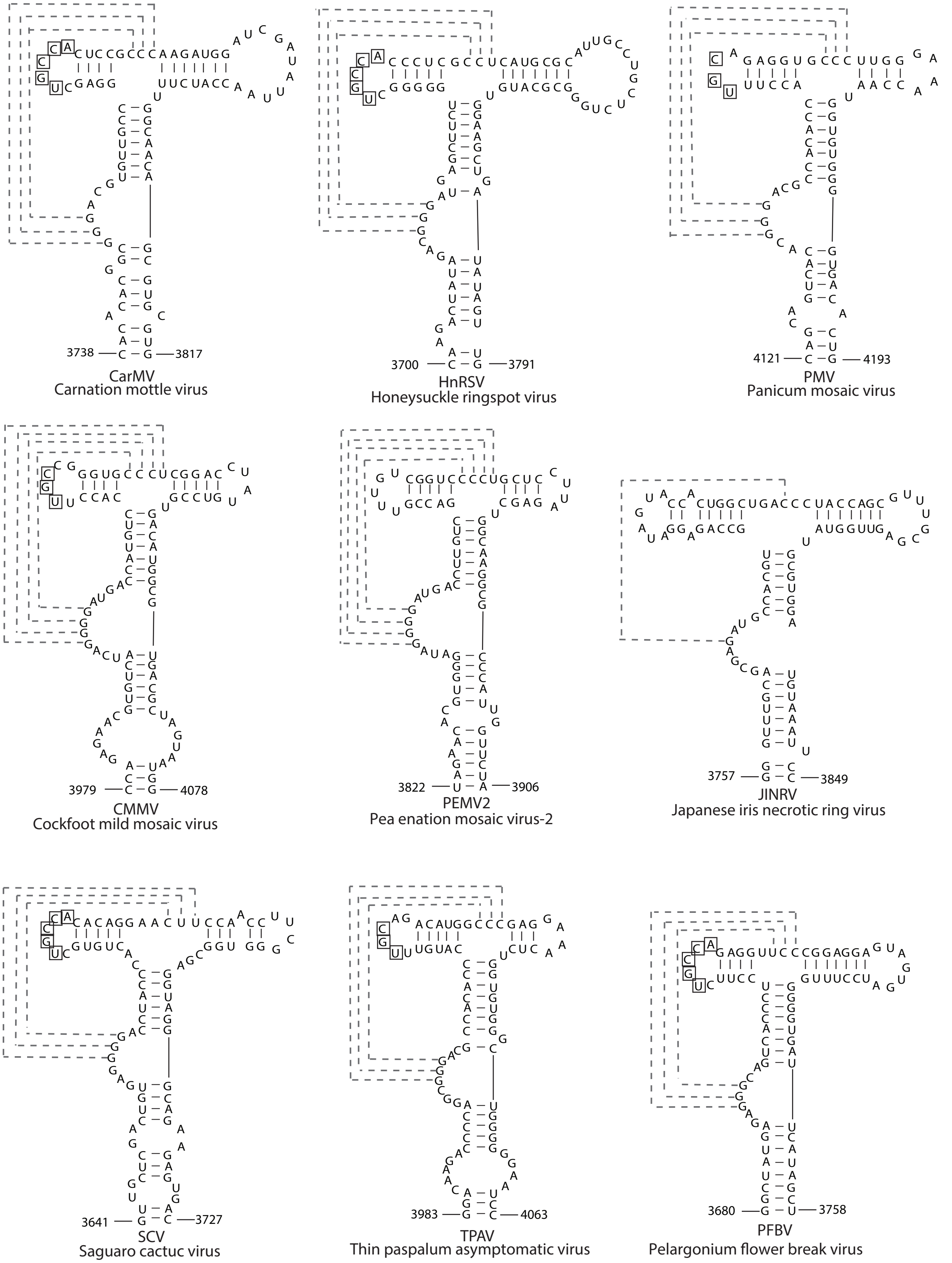

Supplementary Figure S1

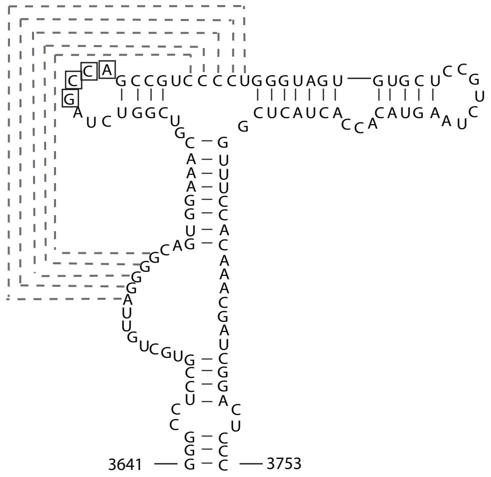

HCRSV
Hibiscus chlorotic ringspot virus

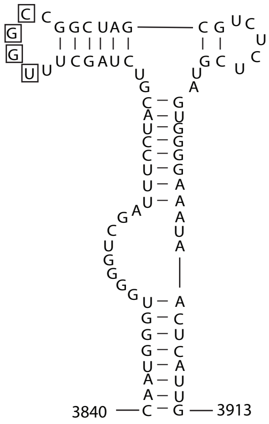

PSNV
Pea stem necrosis virus

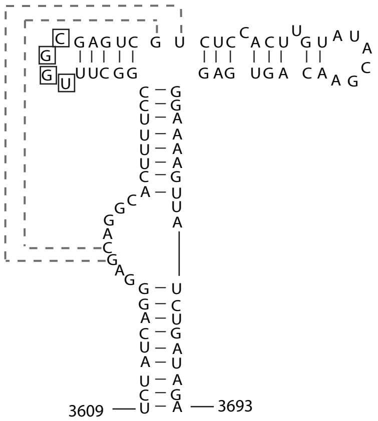

GaMV
Galinsoga mosaic virus

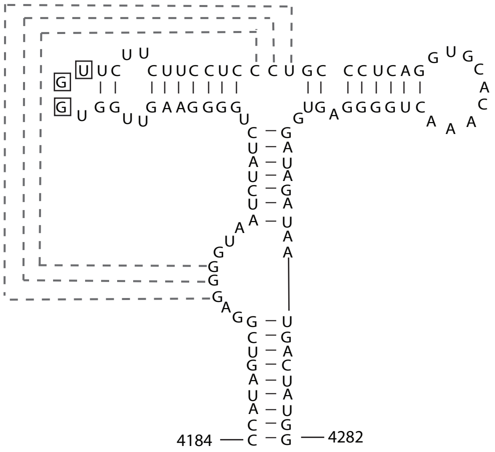

PoLV
Pothos latent virus

Supplementary Figure S1 (Continued)

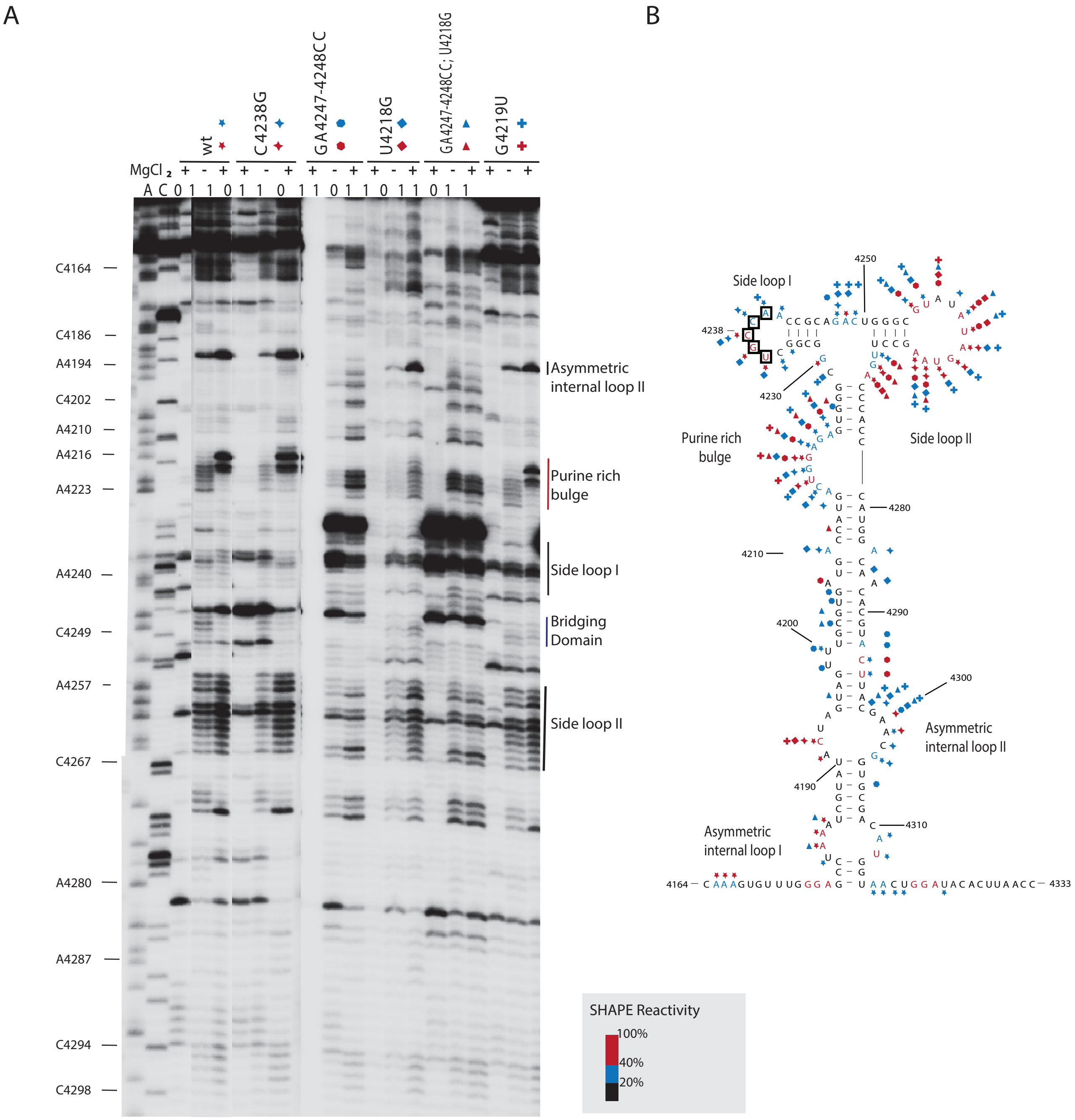

Supplementary Figure S2

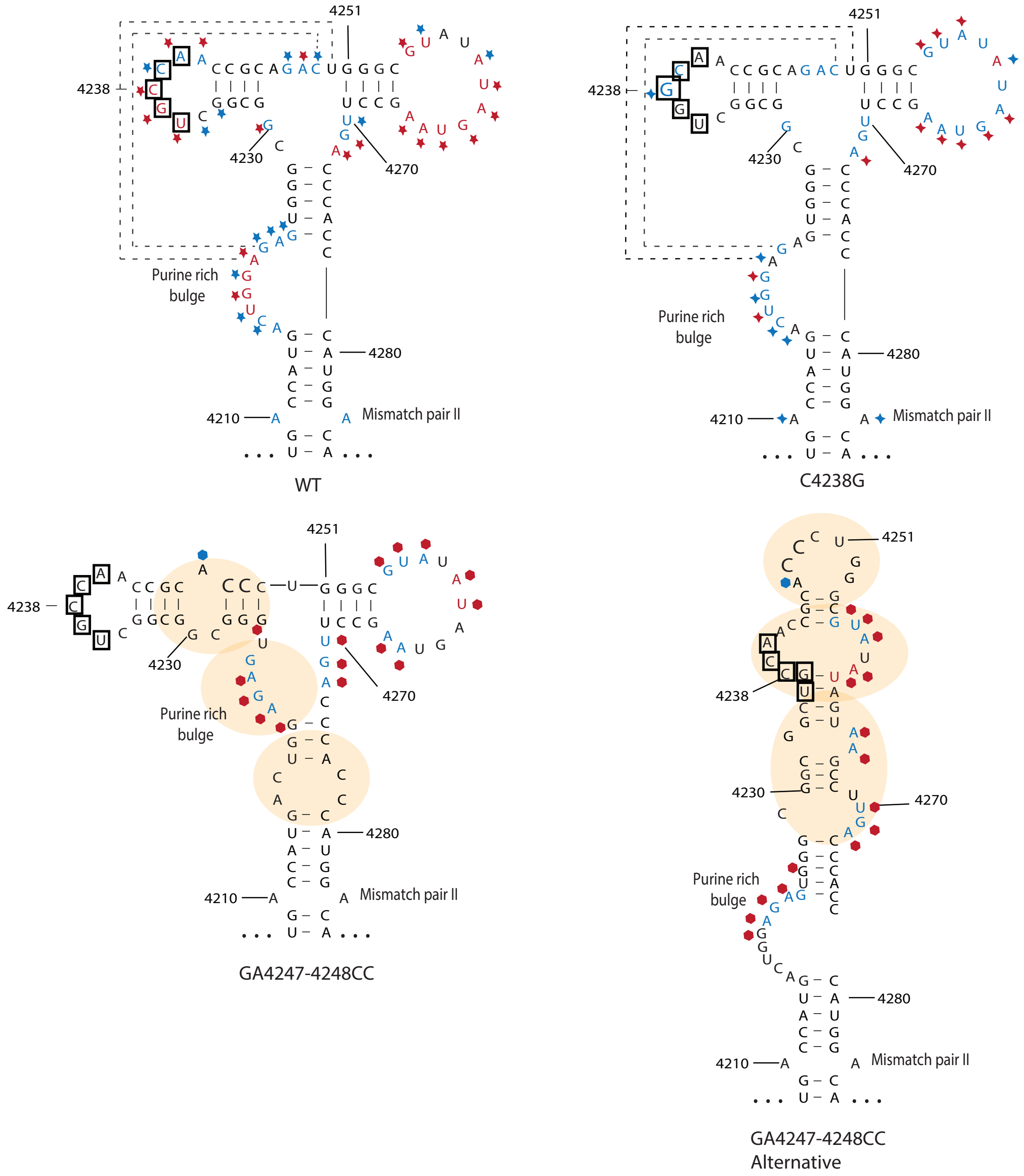

Supplementary Figure S3

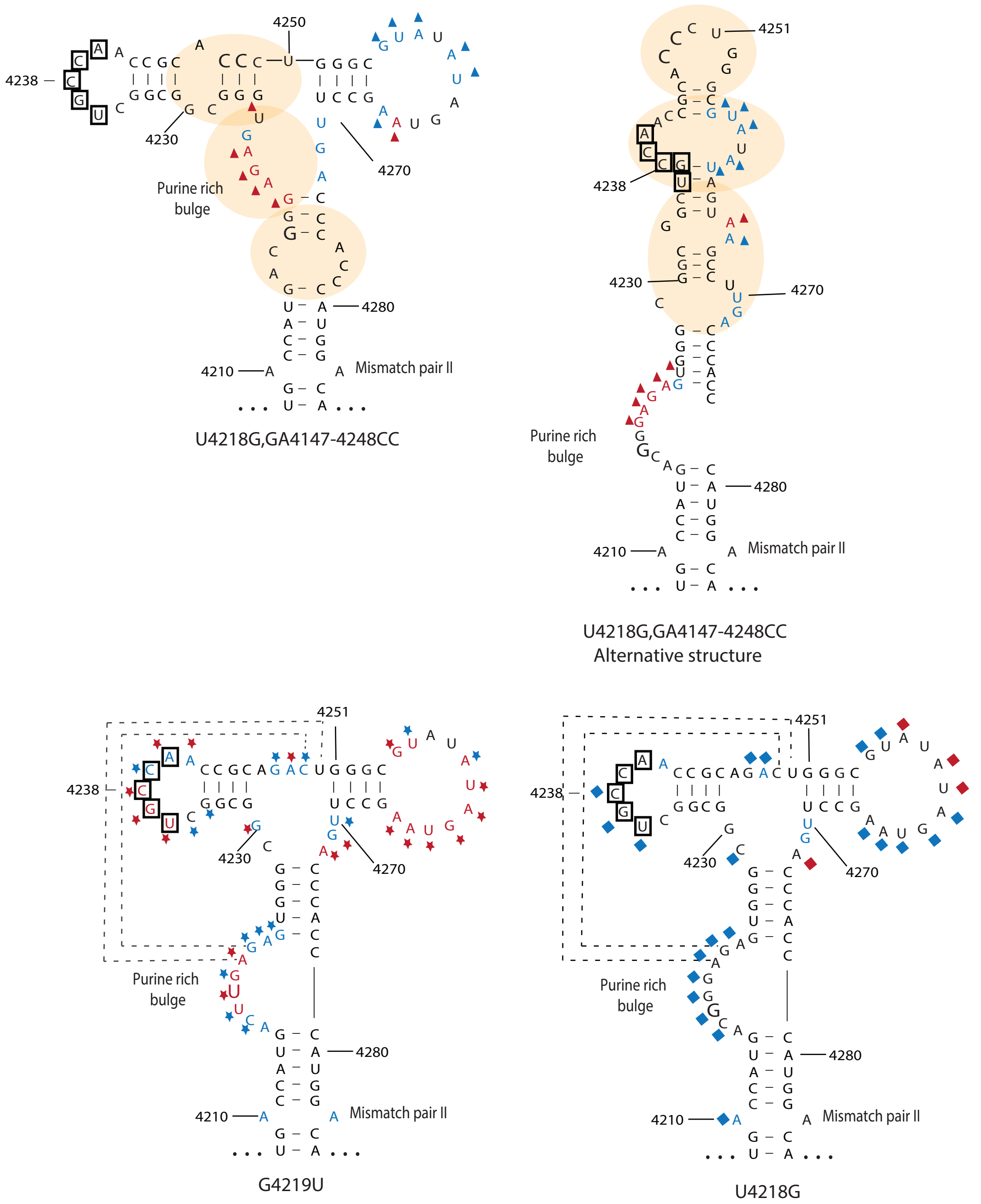

Supplementary Figure S3 (Continued)
